# Upregulation of CCNB2 and a novel lncRNAs-related risk model predict prognosis in clear cell renal cell carcinoma

**DOI:** 10.1007/s00432-024-05611-x

**Published:** 2024-02-01

**Authors:** Congzhe Ren, Qihua Wang, Zhunan Xu, Yang Pan, Shangren Wang, Xiaoqiang Liu

**Affiliations:** Department of Urology, Tianjin Medical University General Hospital, Heping District, 154 Anshan Road, Tianjin, 300052 China

**Keywords:** CCNB2, Clear cell renal cell carcinoma, LncRNA, Prognostic model, Bioinformatics

## Abstract

**Background:**

Clear cell renal cell carcinoma (ccRCC) is the main type of renal cell carcinoma. Cyclin B2 (CCNB2) is a subtype of B-type cyclin that is associated with the prognosis of several cancers. This study aimed to identify the relationship between CCNB2 and progression of ccRCC and construct a novel lncRNAs-related model to predict prognosis of ccRCC patients.

**Methods:**

The data were obtained from public databases. We identified CCNB2 in ccRCC using Kaplan–Meier survival analysis, univariate and multivariate Cox regression, and Gene Ontology analysis. External validation was then performed. The risk model was constructed based on prognostic lncRNAs by the LASSO algorithm and multivariate Cox regression. Receiver operating characteristics (ROC) curves were used to evaluate the model. Consensus clustering analysis was performed to re-stratify the patients. Finally, we analyzed the tumor-immune microenvironment and performed screening of potential drugs.

**Results:**

CCNB2 associated with late clinicopathological parameters and poor prognosis in ccRCC and was an independent predictor for disease-free survival. In addition, CCNB2 shared the same expression pattern with known suppressive immune checkpoints. A risk model dependent on the expression of three prognostic CCNB2-related lncRNAs (SNHG17, VPS9D1-AS1, and ZMIZ1-AS1) was constructed. The risk signature was an independent predictor of ccRCC. The area under the ROC (AUC) curve for overall survival at 1-, 3-, 5-, and 8-year was 0.704, 0.702, 0.741, and 0.763. The high-risk group and cluster 2 had stronger immunogenicity and were more sensitive to immunotherapy.

**Conclusion:**

CCNB2 could be an important biomarker for predicting prognosis in ccRCC patients. Furthermore, we developed a novel lncRNAs-related risk model and identified two CCNB2-related molecular clusters. The risk model performed well in predicting overall survival and immunological microenvironment of ccRCC.

**Supplementary Information:**

The online version contains supplementary material available at 10.1007/s00432-024-05611-x.

## Introduction

Renal cell carcinoma (RCC) is a cancer caused by renal epithelial cells and is one of the most common malignant urological tumors worldwide. There were more than 400,000 new cases of renal cell carcinoma worldwide and more than 170,000 deaths as a result in 2020 (Sung et al. 2021). RCC is divided into three main histologic subgroups: clear cell renal cell carcinoma (ccRCC), papillary renal cell carcinoma (pRCC), and chromophobe renal cell carcinoma (chRCC) (Kovacs et al. 1997). Approximately 70% of RCC patients are diagnosed with ccRCC, the most common and most aggressive RCC subgroup (Motzer et al. 2020). If detected early enough, ccRCC can be treated with surgery or ablation, but one third of patients develop tumor metastasis, which can be fatal (Jonasch et al. 2014). In most cases ccRCC is insensitive to radiotherapy and chemotherapy, and with further research, immunotherapies targeting cytokines or characteristic immune checkpoints have been shown to promote the body’s active immune response through different mechanisms. PD-1/PD-L1 antibodies have been clinically approved for the treatment of metastatic ccRCC (Motzer et al. 2015). However, patients did not respond consistently to immunotherapy (Atkins et al. 2018). Therefore, given the high morbidity and mortality of ccRCC, it is crucial to explore targets with therapeutic as well as prognostic value for the targeted treatment of ccRCC patients.

The cell cycle is a fundamental process of cellular life activity and is tightly regulated by a series of regulatory factors. In the cell cycle, cyclin regulates the activity of cyclin-dependent kinases (CDKs) by binding to CDKs, which allows cells to enter the G2/M phase (Malumbres and Barbacid 2009). Cyclin B2 (CCNB2) belongs to the B-type cyclin family and is an important cell cycle regulator. Abnormal expression of CCNB2 can lead to G2/M checkpoint failure, subsequently resulting in gene mutations, changes in chromosome structure, and even stimulation of tumorigenesis (Yoshitome et al. 2012). CCNB2 has been shown to be highly expressed in a range of human cancers, such as lung cancer, bladder cancer, nasopharyngeal cancer, and liver cancer (Stav et al. 2007; Lei et al. 2016; Gao et al. 2018; Qian et al. 2020). Therefore, CCNB2 may become a promising target for humans in the fight against cancer.

Although the role of CCNB2 in the progression of many cancers is widely investigated, little is known about the effect of CCNB2 on ccRCC. Therefore, we analyzed the expression of CCNB2 in ccRCC and its relationship with clinicopathological factors, and also explored the signaling pathways enriched by CCNB2. The prognostic value of CCNB2 for ccRCC was evaluated.

In addition, it has been shown that the long non-coding RNA (lncRNA)–microRNA (miRNA) axis has an important role in tumor progression and therapeutic resistance. LncRNA has the ability to regulate miRNA function acting as a competitive endogenous RNA (ceRNA) that mediates miRNA sponging and alters expression levels and function (Su et al. 2021). MiRNA reduces gene expression at the post-transcriptional level by binding to the 3′-untranslated region (3′-UTR) of mRNA (Ashrafizadeh et al. 2021). It has been found that in papillary thyroid cancer, MIAT, as the ceRNA of miRNA-150-5p, was significantly and positively correlated with EZH2, the downstream target of miR-150-5p. MIAT may promote invasion of thyroid cancer cells through the miR-150/EZH2 pathway (Guo et al. 2021). According to the available studies, however, CCNB2-related miRNAs or lncRNAs have not been mentioned widely. We then further mined lncRNAs and miRNAs that interact with CCNB2 and constructed a risk model using only three survival-related lncRNAs to reconstitute patients and explore the tumor microenvironment to improve precise treatment.

## Materials and methods

### Patient information acquisition and processing

Gene transcriptomic and clinical data were obtained from The Cancer Genome Atlas (TCGA) (https://tcgadata.nci.nih.gov/tcga/tcgaDownload.jsp) database (Tomczak et al. 2015), including 539 ccRCC samples and 72 normal kidney tissue samples. In addition, both gene expression and clinical data for validation were downloaded from the Gene Expression Omnibus (GEO) (http://www.ncbi.nlm.nih.gov/geo/) database. The construction of risk model was based on TCGA database.

### Association of CCNB2 with various clinical factors and survival

Tumor Immune Estimation Resource 2.0 (TIMER2.0, http://timer.cistrome.org/) was used to explore the differential expression of CCNB2 between tumor tissue and normal tissue (Li et al. 2020). After integrating gene expression data with clinical data, heatmap based on the pheatmap R package was utilized to explore the qualitative relationship between CCNB2 expression and clinical factors. CCNB2 expression between the ccRCC and normal tissues were first compared by *t* test. Subsequently we identified CCNB2 expression of patients in different AJCC stages and ISUP grades by one-way ANOVA test. Considering that gene expression data in GSE53757 were not normalized and were independent of other datasets, we transformed them to log10 (Exp) for ease of processing. Finally, we depicted the relationship between survival status of patients and CCNB2 by the scatter plot using the ggplot2 R package, followed by survival curves using the Kaplan–Meier method on GraphPad Prism 8.0 software with log-rank test. The median value of CCNB2 expression was chosen as the cut-off. *p* < 0.05 was considered statistically significant.

### The Human Protein Atlas

The Human Protein Atlas (https://www.proteinatlas.org) is a program with the aim to map all the human proteins in cells, tissues, and organs using various omics technologies (Asplund et al. 2012). Based on this database, we observed the distribution of CCNB2 protein in ccRCC tissue versus pericancerous tissue by immunohistochemical images.

### Univariate and multivariate Cox regression analysis

CcRCC patients were divided into high and low expression groups according to the mean value of CCNB2 expression. Univariate and multivariate analysis was performed using Cox regression model to find independent prognostic factors, which involved parameters including age, gender (ref. Male), pT stage (ref. T1–T2), pN stage (ref. N0), pM stage (ref. M0), AJCC stage (ref. I–II), ISUP grade (ref. 1–2), and CCNB2 expression (ref. Low expression). The clinical endpoint for patients was set as disease-free survival (DFS). Analysis was performed on IBM SPSS Statistics (version 25.0) and *p* < 0.05 was considered statistically significant.

### Functional enrichment analysis

The set of genes most associated with CCNB2, relying on the Database for Annotation, Visualization and Integrated Discovery (DAVID, v2021) (Huang et al. 2007), was subjected to Gene Ontology (GO) analysis and Kyoto Encyclopedia of Genes and Genomes (KEGG) pathway analysis. The results of the analyses satisfying *p* < 0.05 were arranged in ascending order of *p* value, and the first six results were visualized. The presentation of the results of the GO analysis was based on ggplot2 R package, while the KEGG analysis was presented through Excel (version 2021). To further explore the biological signaling pathways associated with CCNB2, gene set enrichment analysis was performed using GSEA (version 4.3.2) software based on gene expression data from ccRCC patients in the TCGA database, and the results of HALLMARK and KEGG were shown (Subramanian et al. 2005). Significantly enriched gene sets were required to meet Normalized Enrichment Score (NES) > 1, nominal *p* value < 0.05, false discovery rate (FDR) *q* value < 0.05.

### Protein–protein interaction (PPI) network construction

Search Tool for the Retrieval of Interacting Genes (STRING; http://string-db.org) (version 11.5) is a database of known and predicted protein–protein interactions (Franceschini et al. 2013). In this study, an interaction with a combined score > 0.9 was considered statistically significant.

### Correlation analysis of CCNB2 with inhibitory immune checkpoints and immune infiltration

The following analyses were performed on R (version 4.2.1). First, the circlize package was used to visualize the results of the Pearson correlation analysis of CCNB2 with inhibitory immune checkpoints. Subsequently, referring to the leukocyte signature matrix (LM22) file (Newman et al. 2015), we applied CIBERSORT algorithm to obtain the fraction share of immune cells in all ccRCC patient samples. R packages including limma, e1071, parallel and preprocessCore were utilized. Finally, the correlation coefficients between CCNB2 expression and various immune cell infiltrations were obtained with the help of Spearman correlation analysis. An integrated repository portal for tumor-immune system interactions (TISIDB; http://cis.hku.hk/TISIDB/index.php) can be used to demonstrate the relative abundance of 28 tumor-infiltrating lymphocytes (TILs) in a variety of human tumors (Ru et al. 2019).

### The establishment of the predictive nomogram

Patients’ data for constructing nomograms were derived from the TCGA database. With the help of rms R package, the first nomogram was constructed relying on the seven clinicopathological characteristics (gender, age, grade, stage, T, N, and M) and CCNB2 expression to predict disease-free survival (DFS) of ccRCC patients. The second nomogram was constructed based on the age, grade, stage, and risk, predicting overall survival (OS) of patients. Samples lacking related clinicopathological characteristics (gender, age, grade, stage, T, N, and M) were excluded.

### Selection and identification of CCNB2-related miRNAs and lncRNAs

Through RNA Interactome Database (RNAInter; http://www.rna-society.org/rnainter3/) (Lin et al. 2020) and TargetScanHuman (https://www.targetscan.org/vert_80/) (McGeary et al. 2019) databases, we obtained the predicted 70 miRNAs that interacted with CCNB2 (Supplementary Appendix T1). Based on the TCGA database, four miRNAs with a correlation coefficient less than − 0.2 with CCNB2 (*p* < 0.001) were screened by Spearman test. Subsequently 359 predicted lncRNAs binding to these 4 miRNAs were found in the the Encyclopedia of RNA Interactomes (ENCORI; https://starbase.sysu.edu.cn/index.php) (Li et al. 2014), RNAInter, and TargetScanHuman databases (Supplementary Appendix T2). Among them we screened six lncRNAs whose correlation coefficients with CCNB2 were larger than 0.2 (*p* < 0.001).

### Construction and validation of a novel risk model

We randomly divided the patients in TCGA database into train (*n* = 242) and test (*n* = 242) cohorts equally for internal validation. CcRCC patients with an overall survival of less than 30 days were excluded to reduce statistical bias. First, in the train cohort, univariate Cox analysis was performed on six CCNB2-associated lncRNAs to screen for prognostic lncRNAs. To avoid overfitting, least absolute shrinkage and selection operator (LASSO) was performed to further screen prognostic lncRNAs in the train cohort. We then calculated the risk score for each ccRCC patient using the following formula: risk score = gene (*A*) expression × coef (*A*) + gene (*B*) expression × coef (*B*) + … + gene (*i*) expression × coef (*i*) (Shen et al. 2022). Based on the median risk score of train cohort, all patients (*n* = 484) were divided into a high-risk group and a low-risk group. Based on the timeROC R package, we validated the performance of the risk model using receiver operating characteristics (ROC) curves. Subsequently univariate and multivariate Cox analyses of risk scores with clinical factors including age, gender, grade, and stage were performed. Independent prognostic factors were used in the construction of the second nomogram.

### Clusters based on risk signatures

Relying on the expression of lncRNAs in the risk formula, patients were divided into two clusters to further explore the immune microenvironment of patients using the ConsensusClusterPlus R package. Principal component analysis (PCA) and t-distributed stochastic neighbor embedding (t-SNE) were utilized to evaluate the clustering ability of risk signatures.

### Immune infiltration analysis of different risk groups and clusters

Using the “infiltration estimation for tcga” file from the TIMER2.0 database, relying on limma, scales, ggplot2, and ggtext R packages, we performed immune infiltration analyses of patients in risk groups and visualized the results obtained by different methods in a bubble chart. We calculated the immune score for each ccRCC tissue using the estimate R package and compared the differences across risk groups and clusters using the ggpubr R package. The activation of immune checkpoints was also demonstrated by the ggpubr package.

### Pharmacotherapy based on the risk model and clusters

Half-maximal inhibitory concentration (IC50) is the indicator to identify drug sensitivity. R package pRRophetic was used to evaluate therapy response on Genomics of Drug Sensitivity in Cancer (GDSC) (https://www.cancerrxgene.org/) (Yang et al. 2013).

### Statistical analysis

The data were processed using R 4.2.1 software, IBM SPSS Statistics (version 25.0), and GraphPad Prism 8.0. Differential expression levels of CCNB2 between paired stages or grades were compared using Student’s *t* tests. One-way ANOVA test was utilized to measure the overall statistical difference of stages or grades. KM survival analysis was performed by log-rank test. Pearson correlation analysis was used to detect the correlation between CCNB2 and immune checkpoints. The correlation between immune cell infiltration and CCNB2 or risk score was investigated by Spearman’s correlation analysis. We used univariate and multivariate Cox analyses to screen for independent prognostic factors. Unless otherwise stated, *p* < 0.05 was considered statistically significant.

Our study does not contain data from any individual person or any animals.

## Results

### Differential expression of CCNB2 in ccRCC patients

**Fig. 1:**
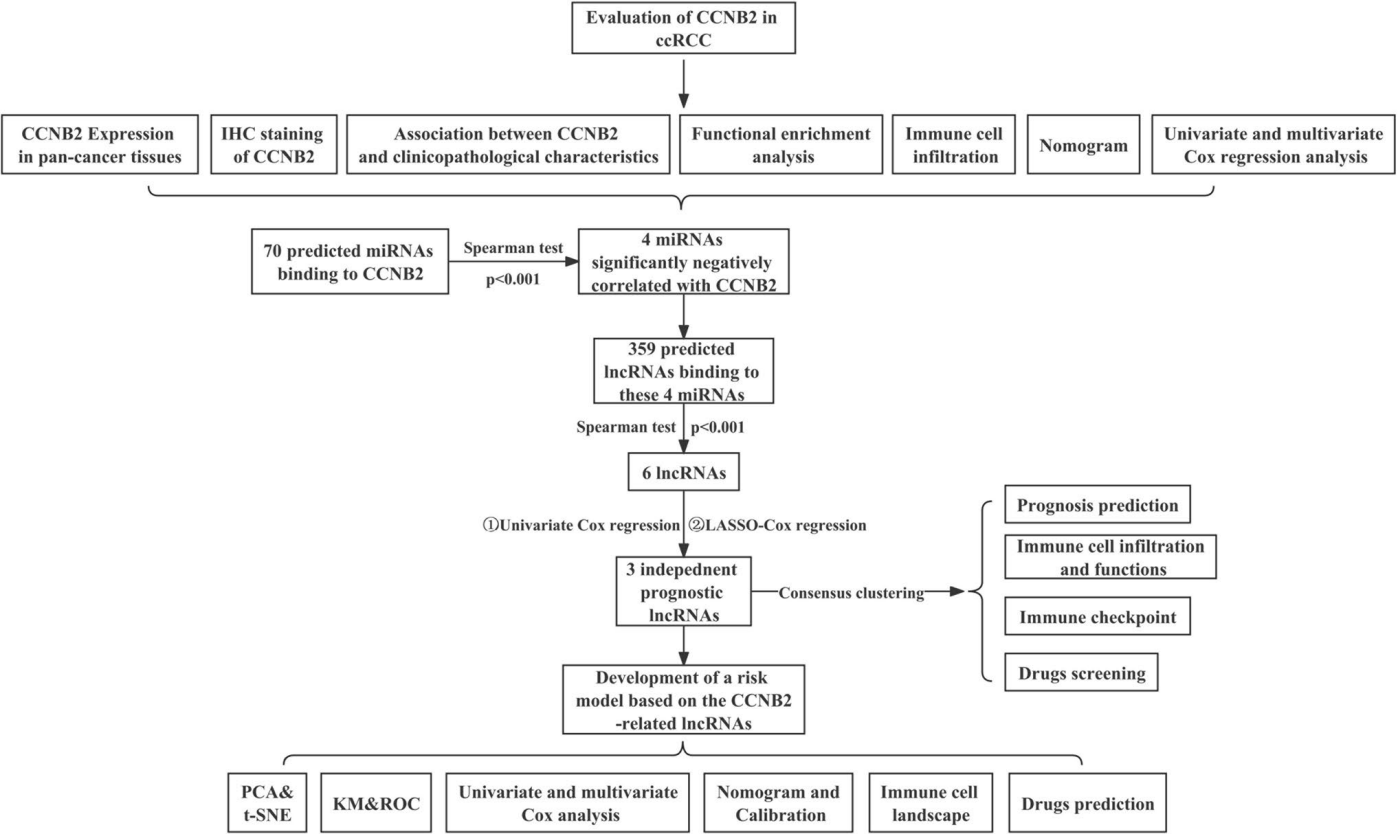
Flow chart of this research

**Fig. 2:**
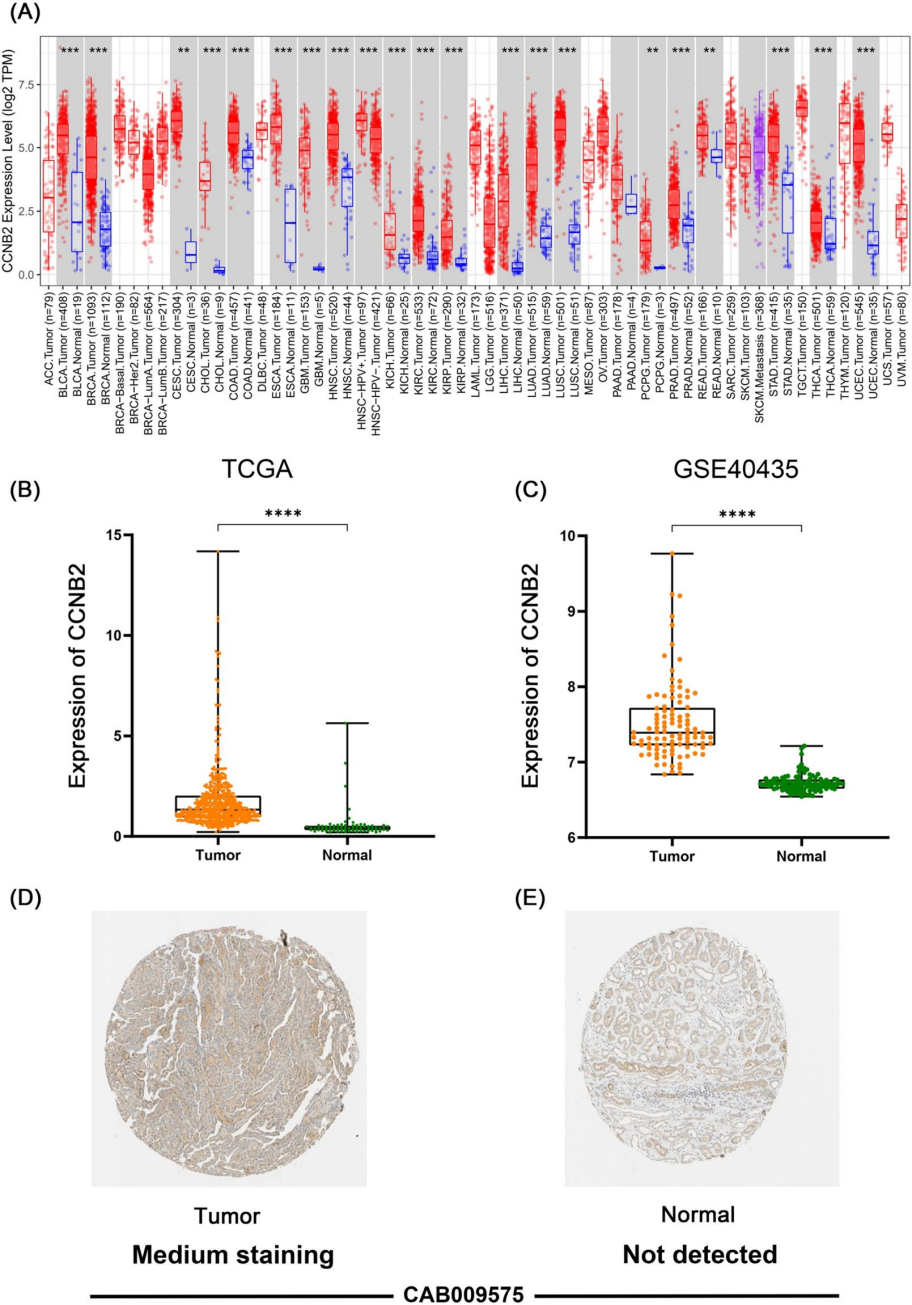
Differential expression of CCNB2 in ccRCC tissues and normal tissues. **A** Differential expression of CCNB2 in a series of tumor tissues and paracancerous tissues based on The Cancer Genome Atlas (TCGA) cohort. **B**, **C** CCNB2 expression was significantly higher in ccRCC tissues than in normal tissues, based on the The Cancer Genome Atlas (TCGA) cohort (****p < 0.0001) and the GSE40435 cohort (****p < 0.0001). **D**, **E** CCNB2 expression was detected in ccRCC tissues but not in normal tissues according to the Human Protein Atlas (HPA) database

The workflow of this study is shown in Fig. 1. Relying on the TIMER 2.0 database, we found that CCNB2 was overexpressed in most tumor tissues, such as bladder cancer (BLCA), breast cancer (BRCA), cervical cancer (CESC), esophageal cancer (ESCA), prostate cancer (PRAD) (*p* < 0.05), and clear cell renal cell carcinoma (ccRCC) (*p* < 0.001) (Fig. 2A). Based on the TCGA database, we found that CCNB2 mRNA was significantly highly expressed in ccRCC tissues relative to normal tissues (*p* < 0.001), which was well validated in the GSE40435 dataset (*p* < 0.001) (Fig. 2B, C).

In addition, IHC staining showed that CCNB2 was undetectably stained in normal kidney tissues (Fig. 2D), while medium levels of expression were observed in ccRCC tissues (Fig. 2E). These results suggested that CCNB2 was highly expressed at transcriptional and proteomic levels in ccRCC tissues.

### High CCNB2 expression is associated with late clinicopathological parameters and poor survival in ccRCC patients

**Fig. 3:**
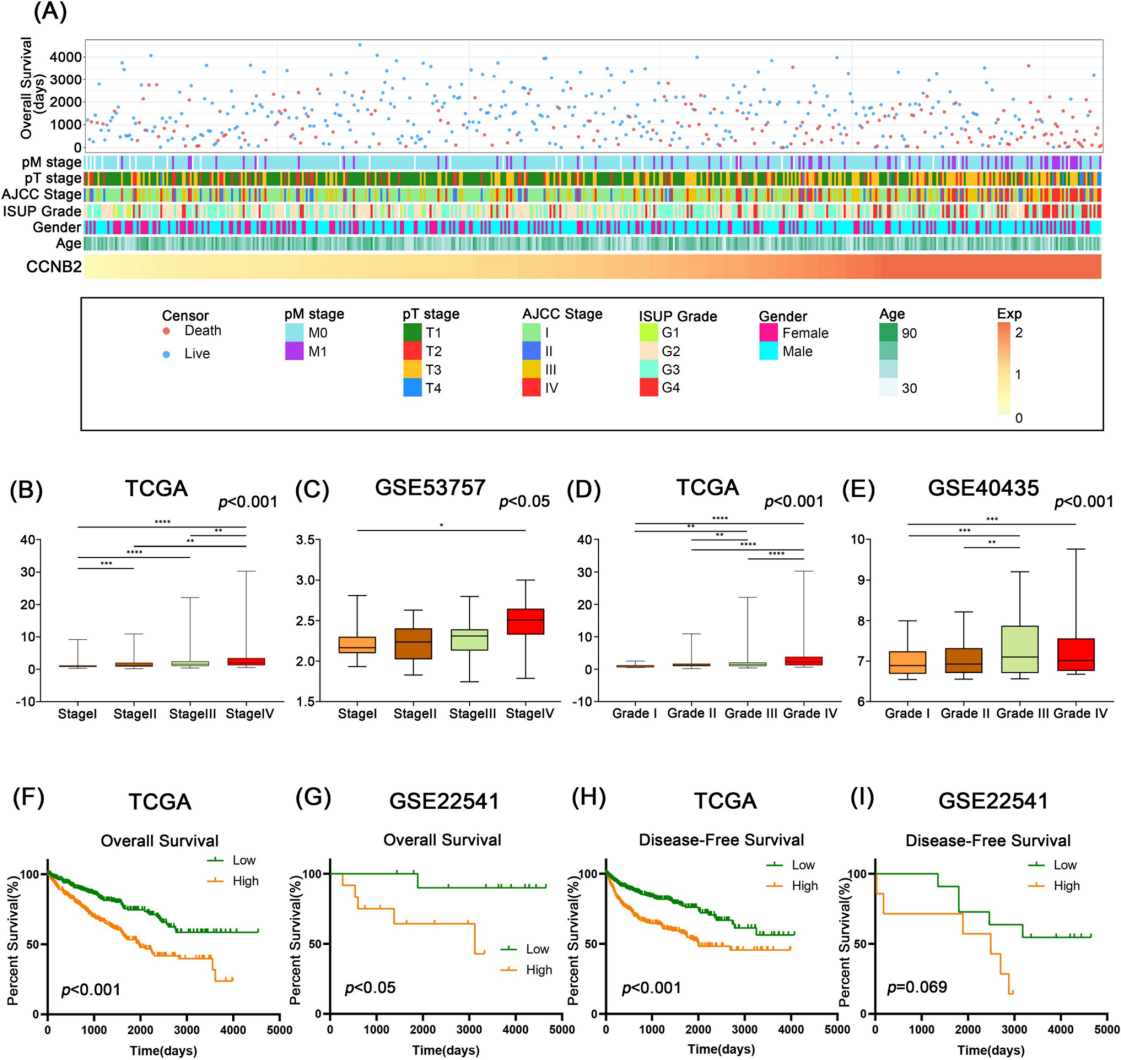
CCNB2 associated significantly with advanced clinicopathological factors and poor survival in ccRCC patients. **A** The heatmap of CCNB2-related clinicopathological factors of ccRCC in the TCGA cohort. **B**, **C** CCNB2 expression was significantly increased in ccRCC with advanced stage in the TCGA and GSE53757 cohorts. **D**, **E** CCNB2 expression was significantly correlated with ISUP grade in the TCGA and GSE40435 cohorts, with more advanced patients tending to have higher CCNB2 expression. *p < 0.05, **p < 0.01, ***p < 0.001, ****p < 0.0001. **F**, **G** Kaplan–Meier analysis showed that CCNB2 was significantly associated with shorter overall survival in the TCGA (*p* < 0.001) and GSE22541 cohorts (*p* < 0.05). **H**, **I** CCNB2 was associated with shorter disease-free survival (DFS) in the TCGA (*p* < 0.001) and GSE22541 cohorts (*p* = 0.069)

Patients with different CCNB2 expression presented with different clinicopathological features. With the increase of CCNB2 expression, AJCC stage, IUSP grade, pT stage, pM stage, and OS showed asymmetric distribution in the TCGA dataset (Fig. 3A). Based on the clinicopathological data from the TCGA dataset, we found that CCNB2 mRNA expression in ccRCC samples increased with increasing stage level (*p* < 0.001) (Fig. 3B). Similarly, CCNB2 expression was also correlated with pathological grade (*p* < 0.001) (Fig. 3D). The above results were validated, respectively, in the GSE53757 dataset (*p* < 0.05) and the GSE40435 dataset (*p* < 0.001) (Fig. 3C, E). Overall, these results suggested that ccRCC with higher malignancy was enriched with CCNB2.

To investigate the effect of CCNB2 on the prognosis of ccRCC patients, we performed Kaplan–Meier survival analysis and Cox regression analysis. In the TCGA dataset, patients with high CCNB2 expression had significantly shorter OS (*p* < 0.001) and DFS (*p* < 0.001) (Fig. 3F, H). Meanwhile, we validated the relationship between CCNB2 expression and OS in the GSE22541 dataset (*p* < 0.05) (Fig. 3G). Although the association of CCNB2 expression with shorter DFS was not statistically significant in the GSE22541 dataset (*p* = 0.069), it showed the same trend (Fig. 3I).

### CCNB2 is an independent prognostic factor for disease-free survival (DFS) in ccRCC patients

AJCC stage, pTNM stage, ISUP grade, and gender were associated with DFS in the univariate Cox regression analysis model (*p* < 0.05), and high CCNB2 expression (HR = 3.011, *p* < 0.001) was significantly associated with lower DFS (Table 1). More importantly, in the multivariate Cox regression analysis model, high CCNB2 expression (HR = 2.114, *p* < 0.001), pM stage (HR = 3.995, *p* < 0.001), and AJCC stage (HR = 2.824, *p* < 0.05) remained markedly associated with poor DFS (Table 1). Except for age, pN stage, and ISUP grade, all other variables were validated in the GSE22541 dataset, especially CCNB2 expression (univariate: HR = 3.607, *p* < 0.05; multivariate: HR = 3.357, *p* < 0.05) (Table 2). These results suggested that CCNB2 could serve as an independent predictor of prognosis in ccRCC, and high CCNB2 expression indicated a poor prognosis for patients.

**Table 1:** Univariate and multivariate Cox regression analysis of disease-free survival (DFS) in The Cancer Genome Atlas (TCGA) cohort

Variable	Univariate analysis		Multivariate analysis	
	HR (95% CI)	*p* value	HR (95% CI)	*p* value
CCNB2 (ref. low expression)	3.011 (2.192–4.137)	*1.03e−11*	2.114 (1.282–3.486)	*0.003*
Age	1.007 (0.994–1.020)	0.294	–	–
Gender (ref. female)	1.481 (1.041–2.107)	*0.029*	1.25 (0.795–1.964)	0.334
pT stage (ref. T1–T2)	4.539 (3.263–6.314)	*2.65e−19*	1.121 (0.534–2.35)	0.763
pN stage (ref. N0)	3.860 (1.979–7.530)	*7.40e−05*	0.971 (0.47–2.005)	0.936
pM stage (ref. M0)	8.655 (6.211–12.060)	*3.16e−37*	3.995 (2.318–6.884)	*6.09e−07*
AJCC stage (ref. I–II)	6.730 (4.685–9.668)	*5.96e−25*	2.824 (1.121–7.117)	*0.028*
ISUP grade (ref. 1–2)	3.370 (2.309–4.918)	*2.97e−10*	1.414 (0.829–2.411)	0.204

*p*-values lower than 0.05 are displayed in italics

**Table 2:** Univariate and multivariate Cox regression analysis of disease-free survival (DFS) in GSE22541 cohort

Variable	Univariate analysis		Multivariate analysis	
	HR (95% CI)	*p* value	HR (95% CI)	*p* value
CCNB2 (ref. low expression)	3.607 (1.294–10.057)	*0.014*	3.357 (1.087–10.364)	*0.035*
Age	–	–	–	–
Gender (ref. female)	1.234 (0.469–3.250)	0.670	–	–
pT stage (ref. T1–T2)	1.281 (0.416–3.944)	0.666	–	–
pN stage (ref. N0)	–	–	–	–
pM stage (ref. M0)	9.740 (1.797–52.788)	*0.008*	4.522 (0.719–28.433)	0.108
AJCC stage (ref. I–II)	2.784 (1.042–7.440)	*0.041*	2.21 (0.646–7.56)	0.206
ISUP grade (ref. 1–2)	–	–	–	–

*p*-values lower than 0.05 are displayed in italics

### Functional annotations and signaling pathways

**Fig. 4:**
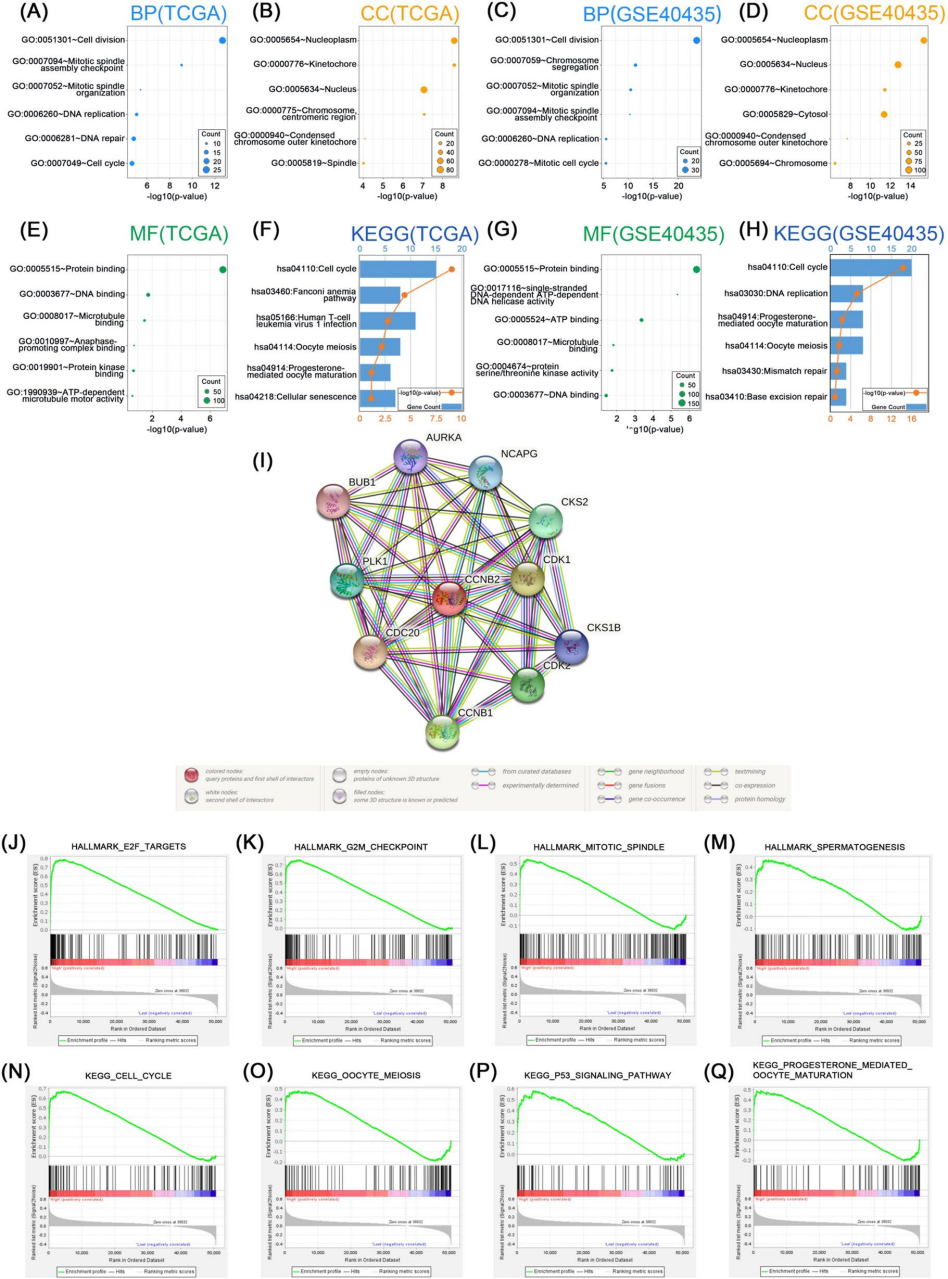
Functional enrichment and signaling pathways. **A**, **B**, **E** Biological processes (BP), cellular components (CC), and molecular functions (MF) mostly related to CCNB2 in the TCGA database. (F) Kyoto Encyclopedia of Genes and Genomes (KEGG) pathway analysis of CCNB2 in the TCGA database. **C**, **D**, **G** Biological processes (BP), cellular components (CC), and molecular functions (MF) mostly related to CCNB2 in the GSE40435 data set. **H** Kyoto Encyclopedia of Genes and Genomes (KEGG) pathway analysis of CCNB2 in the GSE40435 cohort. **I** The protein–protein interaction (PPI) network of CCNB2 and the co-expressed proteins was constructed visually. **J**–**Q** Gene set enrichment analysis (GSEA) showed CCNB2 was enriched in signaling pathways, including E2F targets, G2M checkpoint, mitotic spindle, spermatogenesis, cell cycle, oocyte meiosis, p53 signaling pathway and progesterone-mediated oocyte maturation

To explore the biological functions of CCNB2, we did functional enrichment analyses. Genes associated with CCNB2 were screened by Pearson correlation analysis (|Cor|> 0.5, *p* < 0.05) for GO (BP: biological process, CC: cellular component, MF: molecular function) and KEGG analysis. The biological processes most relevant to CCNB2 in the TCGA database were cell division, mitotic spindle assembly checkpoint, mitotic spindle organization, and DNA replication (Fig. 4A). CCNB2 was mainly distributed in nucleoplasm, kinetochore, and nucleus (Fig. 4B). In addition, the molecular functions of CCNB2 were significantly correlated with protein binding, DNA binding, and microtubule binding (Fig. 4E). According to the results of KEGG analysis, the most relevant signaling pathway was cell cycle, in addition to oocyte meiosis and progesterone-mediated oocyte maturation (Fig. 4F). GO and KEGG analyses demonstrated similar results in the GSE40435 dataset (Fig. 4C, D, G, H). These findings suggested that CCNB2 might play an important role in the progression of ccRCC by affecting the cell cycle, interfering with the normal cell division process and causing cells to develop a pro-cancer phenotype. Furthermore, we constructed a PPI network for CCNB2 in which ten genes (CCNB1, CDC20, PLK1, BUB1, AURKA, NCAPG, CKS1B, CKS2, CDK1, CDK2) were meaningfully associated with the expression of CCNB2 (Fig. 4I). To further validate and explore the signal pathways associated with CCNB2, GSEA was performed and eight signaling pathways were identified, including E2F targets, G2M checkpoint, mitotic spindle, spermatogenesis, cell cycle, oocyte meiosis, p53 signaling pathway, and progesterone-mediated oocyte maturation. The expression of CCNB2 was significantly enriched in these signaling pathways (*p* value < 0.001, FDR < 0.25) (Fig. 4J–Q).

### High CCNB2 expression positively correlates with activation of inhibitory immune checkpoints and affects immune infiltration

**Fig. 5:**
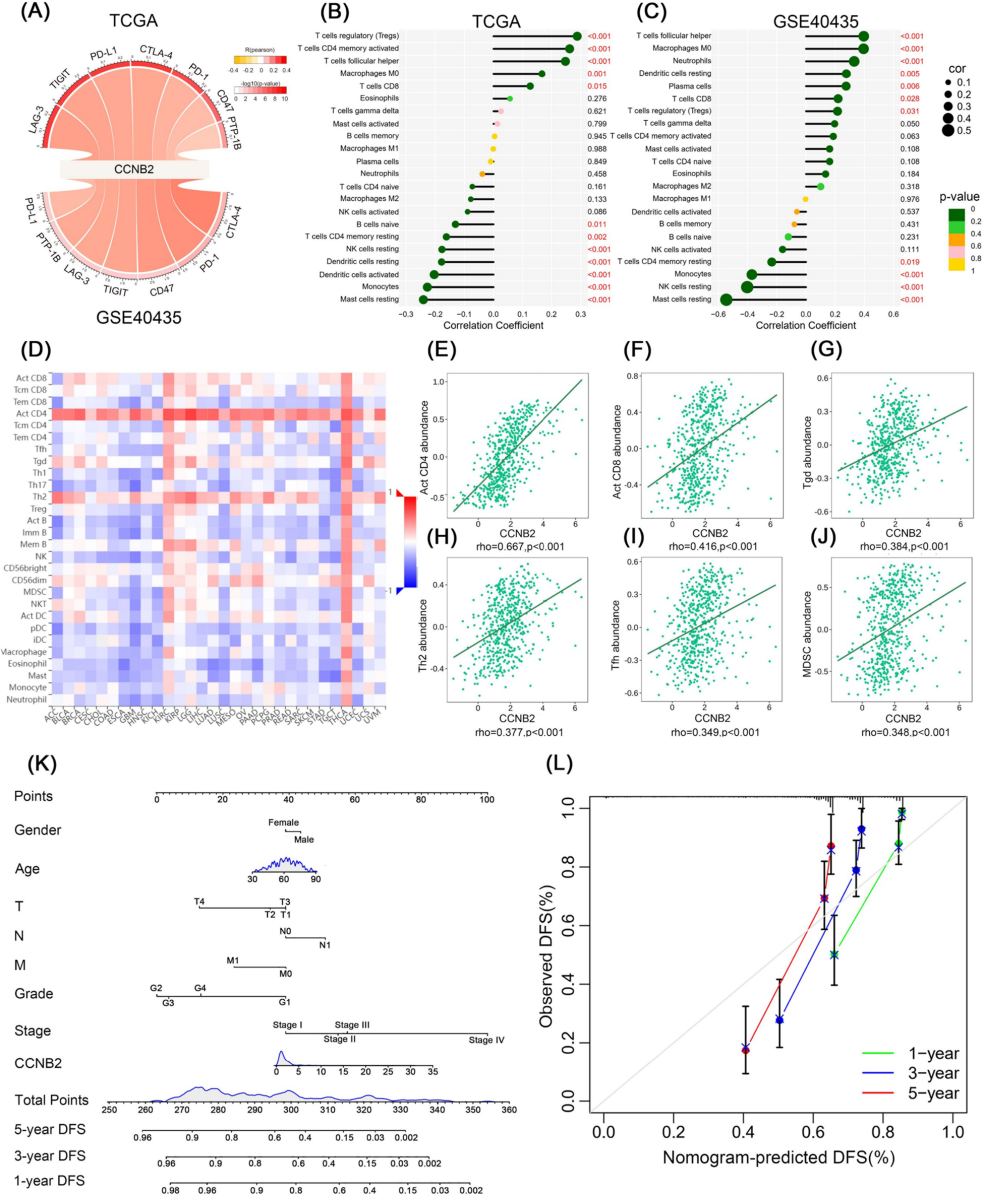
Correlation of CCNB2 expression with ccRCC immune infiltration and establishment of a nomogram model. **A** Pearson correlation analysis between CCNB2 and known suppressive immune checkpoints. The width of the strip represented the correlation coefficient (*R*). *p* value was indicated by the color of the strip. **B**, **C** Correlation of CCNB2 expression with immune infiltration in ccRCC patients in the TCGA and GSE40435 datasets based on the CIBERSORT algorithm. The size of the sphere represents the correlation coefficient. The color of the sphere represents the *p* value. **D** Association between the expression of CCNB2 and a series of tumor-infiltrating lymphocytes (TILs) in tumors. **E**–**J** CCNB2 was significantly relevant to abundance of activated CD4 T cells (Act CD4 cells), activated CD8 T cells (Act CD8 cells), gamma delta T cells (Tgd cells), type 2 T helper cells (Th2 cells), T follicular helper cells (Tfh cells), and myeloid-derived suppressor cells (MDSC). **K** Nomogram was used to predict 1-, 3-, 5-year disease-free survival (DFS) of ccRCC patients in the TCGA database. **L** The calibration curve of nomogram

To explore the association between CCNB2 and immunosuppression of ccRCC, we selected seven known inhibitory immune checkpoints including LAG-3, TIGIT, PD-1, PD-L1, CTLA-4, CD47, and PTP-1B. Pearson correlation analysis demonstrated a significant positive correlation between CCNB2 and the activation of these inhibitory immune checkpoints in the TCGA and GSE40435 datasets (*p* < 0.05), leading to suppression of the immune response in ccRCC (Fig. 5A). After clarifying the relationship between CCNB2 and inhibitory immune checkpoints, we proceeded to analyze the effect of CCNB2 on tumor-immune infiltration. As Fig. 5D demonstrates, CCNB2 was significantly associated with 28 TILs in a variety of human cancers. In renal clear cell carcinoma and thyroid cancer, elevated CCNB2 led to a general increase in immune infiltration. Moreover, CCNB2 increased the infiltration of activated CD4 T cells (Act CD4 cells) and type 2T helper cells (Th2 cells) in most tumors (Fig. 5D). CCNB2 was significantly correlated with important TILs in ccRCC, including activated CD4 T cells (Act CD4 cells; *ρ* = 0.667, *p* < 0.001), activated CD8 T cells (Act CD8 cells; *ρ* = 0.416, *p* < 0.001), gamma delta T cells (Tgd cells; *ρ* = 0.384, *p* < 0.001), type 2T helper cells (Th2 cells; *ρ* = 0.377, *p* < 0.001), T follicular helper cells (Tfh cells; *ρ* = 0.349, *p* < 0.001), and myeloid-derived suppressor cells (MDSC; ρ = 0.348, *p* < 0.001) (Fig. 5E–J).

Subsequently, we did further CIBERSORT analysis. It is known from Fig. 5B that infiltration of several immune cells in ccRCC samples was significantly and positively associated with high CCNB2 expression, including T regulatory cells (Tregs), activated memory CD4 T cells, T follicular helper cells, M0 macrophages, and CD8 T cells (*p* < 0.05), which was verified in the GSE40435 dataset (Fig. 5C). Conversely, CCNB2 also showed a negative correlation with the infiltration of some immune cells in ccRCC samples, including resting mast cells, monocytes, and resting memory CD4 T cells (*p* < 0.05) (Fig. 5B, C).

### A nomogram model was established to assess the prognosis of ccRCC patients

To assess the prognosis of ccRCC, a nomogram was constructed to predict the DFS of patients based on seven clinicopathological characteristics (gender, age, grade, stage, T, N, and M) and CCNB2 expression. The different clinicopathological factors of ccRCC patients were converted into scores according to the scale in the nomogram, and the scores of the different factors were summed to obtain a total score. We were also able to predict 1-, 3-, and 5-year DFS based on the total score (Fig. 5K). In addition, we made the calibration chart (Fig. 5L), and the results showed that the nomogram could predict the DFS of ccRCC patients well, which allowed for more accurate prediction of prognosis for ccRCC patients.

### CCNB2-related miRNAs and lncRNAs and the establishment and validation of the model

**Fig. 6:**
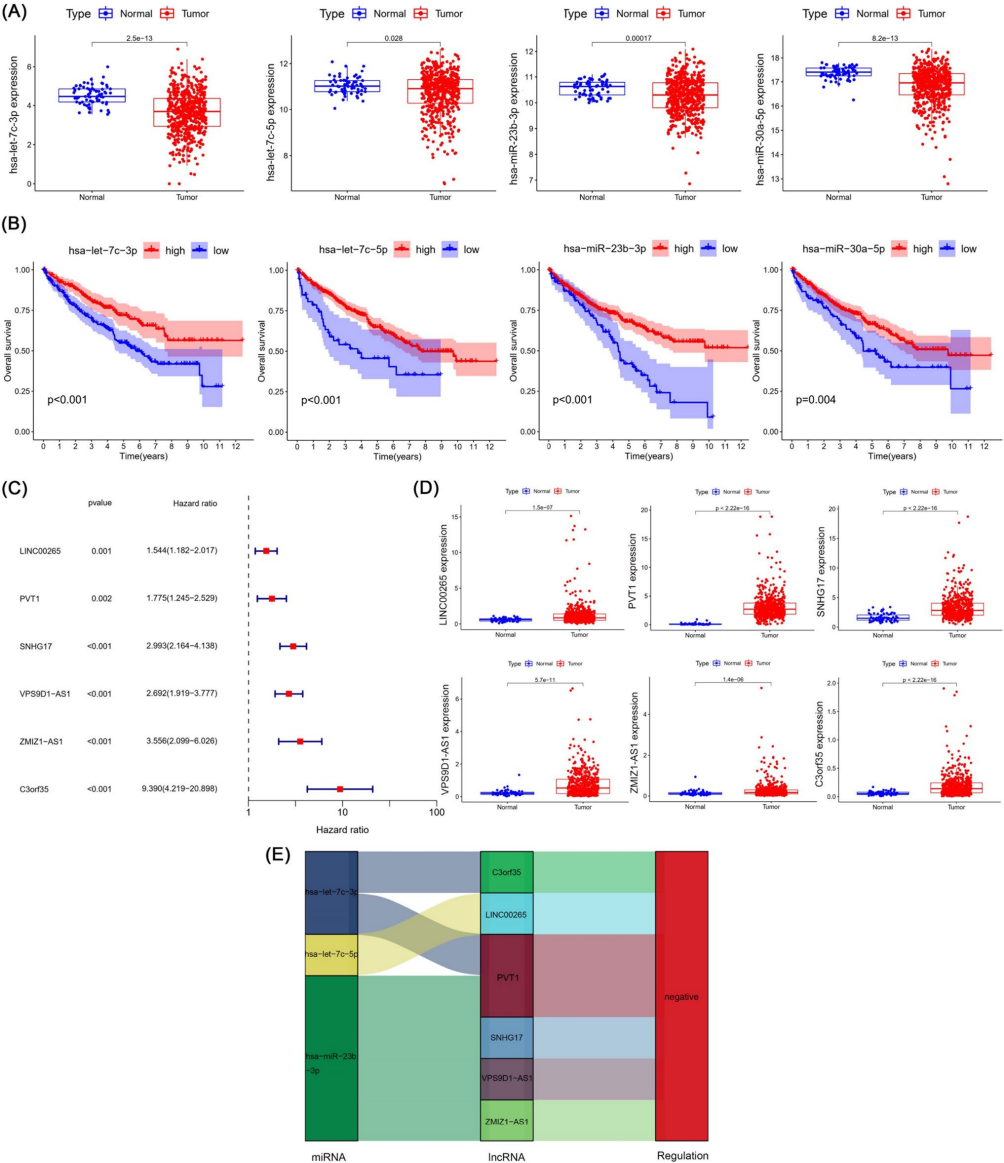
CCNB2-related prognostic miRNAs and lncRNAs in ccRCC. **A**, **B** The four miRNAs screened were lowly expressed in ccRCC and correlated with prognosis. **C** The prognostic lncRNAs screened by univariate Cox regression analysis. **D** The prognostic lncRNAs were enriched in ccRCC tissues. **E** Regulatory network of CCNB2-related miRNAs and lncRNAs

**Fig. 7:**
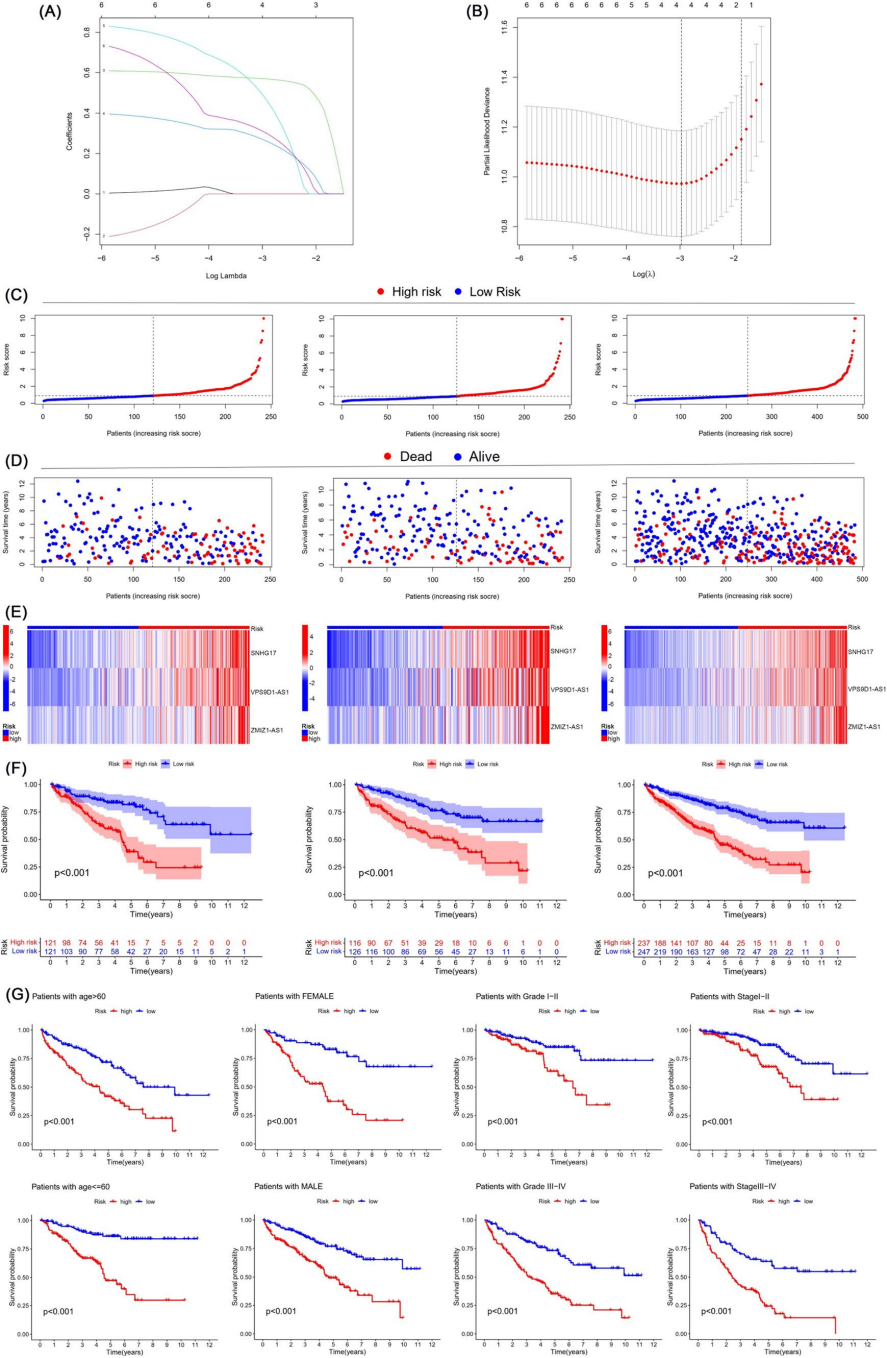
Construction of the risk signature relying on three CCNB2-related lncRNAs. **A** LASSO coefficient profiles of six prognostic CCNB2-related lncRNAs. **B** Profiles of LASSO deviance. **C** Risk score plots in the train, test, and entire cohorts. **D** Survival status plots between low-risk and high-risk groups in the train, test, and entire cohorts. **E** The heatmap of three CCNB2-related lncRNAs expression in the train, test, and entire cohorts. **F** Kaplan–Meier survival curves of overall survival between low-risk and high-risk groups in the train, test, and entire cohorts. **G** Kaplan–Meier survival curves of overall survival were validated in different stratifications including age, gender, grade and stage between low-risk and high-risk groups in the entire cohort

**Fig. 8:**
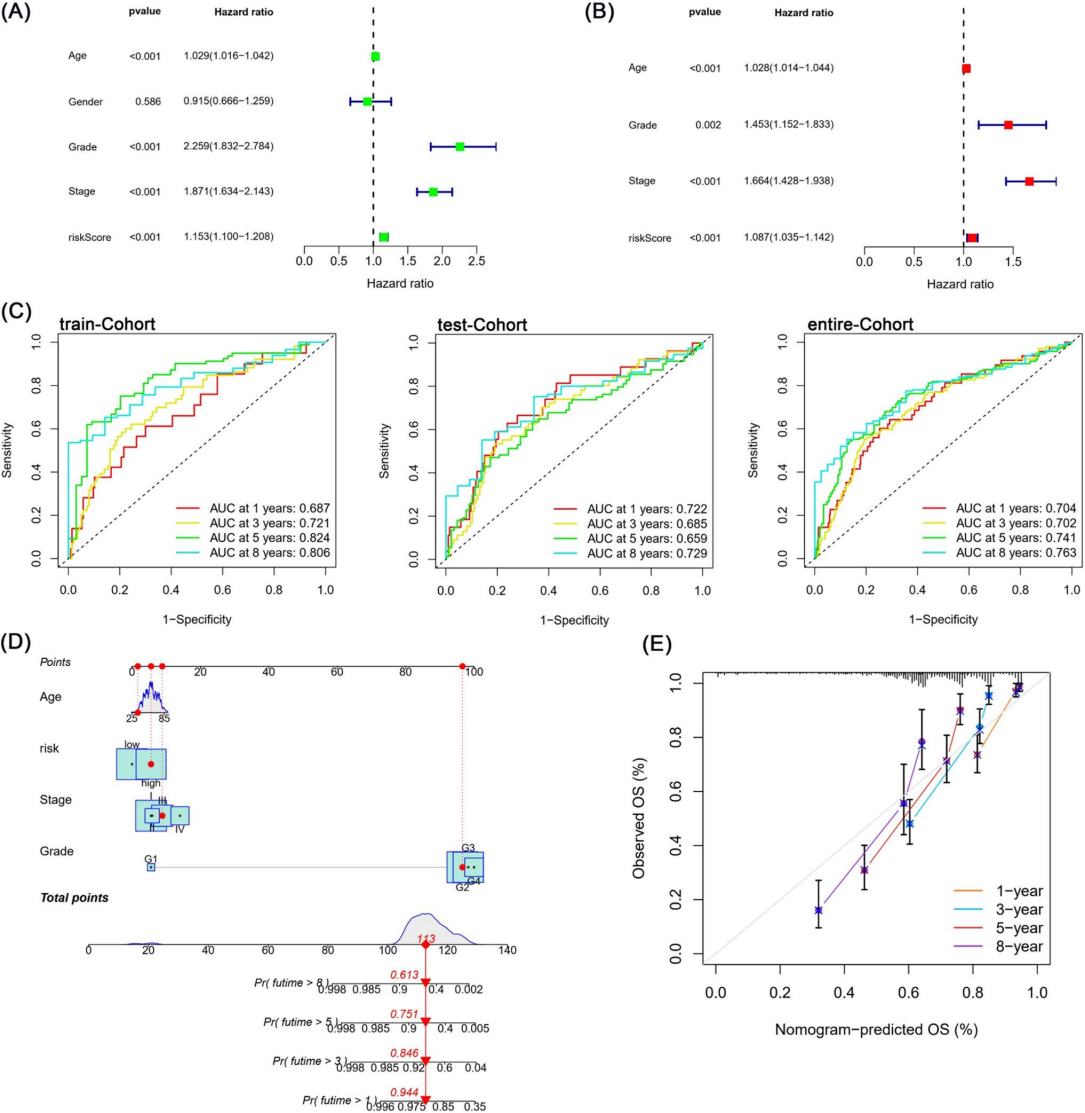
Evaluation of the risk model and construction of the nomogram. **A**, **B** Univariate and multivariate Cox regression analyses for overall survival of ccRCC patients. **C** The 1-, 3-, 5-, and 8-year receiver operating characteristic (ROC) curves of the risk model in the train, test, and entire cohorts. **D** The nomogram based on the independent prognostic factors including age, grade, stage, and risk predicted the 1-, 3-, 5-, and 8-year overall survival. **E** Time-dependent calibration curves for 1-, 3-, 5-, and 8-year overall survival

Four miRNAs that were significantly negatively correlated with CCNB2 expression were all reduced in tumors (Fig. 6A), and their low expression was significantly associated with poor prognosis in ccRCC patients (Fig. 6B), suggesting that these miRNAs may have a diminished regulatory effect on CCNB2 expression in tumors resulting in a poor prognosis. Subsequently, six predicted lncRNAs that interacted with the above four miRNAs were mined out and subjected to univariate Cox analysis, all of which were prognostic risk factors for ccRCC patients, including LINC00265, PVT1, SNHG17, VPS9D1-AS1, ZMIZ1-AS1, and C3orf35 (Fig. 6C) and were enriched in ccRCC (Fig. 6D). The regulation relationship between CCNB2-related miRNAs and lncRNAs was demonstrated in the Sankey diagram (Fig. 6E). To avoid overfitting, lasso regression analysis was performed on these six lncRNAs, and the four most significant lncRNAs were extracted when the likelihood deviance was minimal, including SNHG17, VPS9D1-AS1, ZMIZ1-AS1, and C3orf35 (Fig. 7A, B). They were used to construct multivariate Cox model and obtain the equation for the risk score: risk score = SNHG17 × (0.6518) + VPS9D1-AS1 × (0.4443) + ZMIZ1-AS1 × (0.8056). Risk score, survival status, and distribution of risk genes were compared between the high- and low-risk groups based on the risk score formula in the train, test, and entire cohorts (Fig. 7C–E). Kaplan–Meier survival curves showed that the high-risk group had lower overall survival (*p* < 0.001) (Fig. 7F), which was verified in cohorts of different age, sex, grade, and stage (Fig. 7G). Time-dependent receiver operating characteristic (ROC) curves were used to validate the ability of the risk model to predict the prognosis of ccRCC patients (Fig. 8C). The area under curve (AUC) for 1-, 3-, 5-, and 8-year was 0.687, 0.721, 0.824, and 0.806 for the train cohort, 0.722, 0.685, 0.659, and 0.729 for the test cohort, and 0.704, 0.702 0.741, and 0.763 for the entire cohort, respectively.

### Establishment of a risk-related nomogram

In the univariate regression model, risk score (HR = 1.153, *p* < 0.001), stage (HR = 1.87, *p* < 0.001), grade (HR = 2.259, *p* < 0.001), and age (HR = 1.029, *p* < 0.001) were significantly associated with overall survival of patients (Fig. 8A). More importantly, the risk score (HR = 1.087, *p* < 0.001) remained significantly associated with overall survival in the multivariate regression model. Besides, we also found three other independent prognostic factors, age (HR = 1.028, *p* < 0.001), grade (HR = 1.453, *p* = 0.002), and stage (HR = 1.664, *p* < 0.001) (Fig. 8B). Based on these four independent prognostic factors, we constructed a nomogram predicting overall survival at 1-, 3-, 5-, and 8-year for ccRCC patients (Fig. 8D). The calibration plot demonstrated that the nomogram could well predict the overall survival of patients at 1-, 3-, 5-, and 8-year (Fig. 8E).

### Immune infiltration and immunotherapy in the risk group

**Fig. 9:**
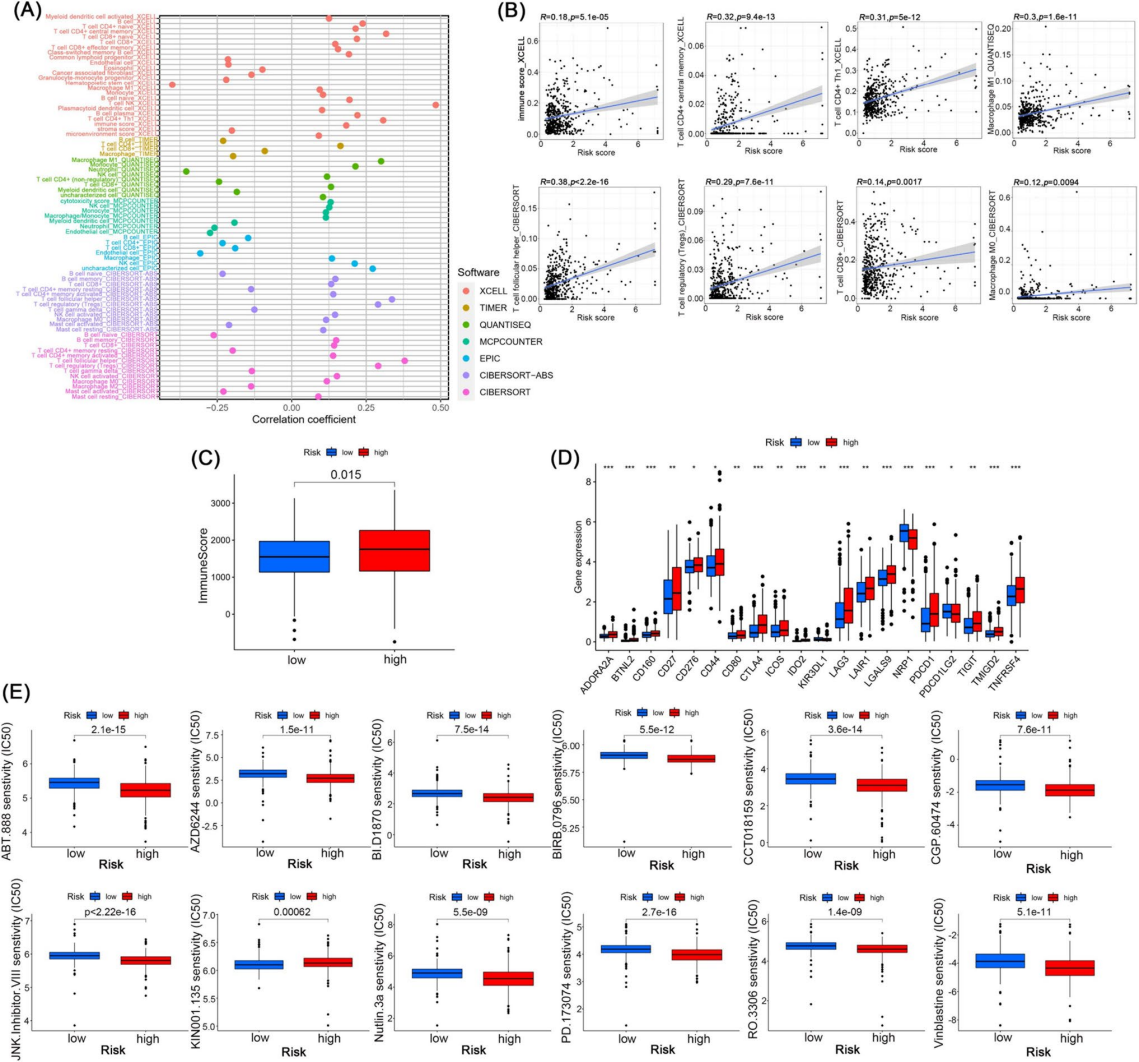
Association between risk signature and immune cell infiltration. **A** Immune cell infiltration levels in different platforms in ccRCC patients. **B** Relationship between risk score and immune cells. **C** Immune score between low-risk and high-risk groups. **D** Activation of immune checkpoints between low-risk and high-risk groups, *p < 0.05, **p < 0.01, ***p < 0.001, ****p < 0.0001. **E** Immunotherapy sensitivity analysis of risk groups

As demonstrated in Fig. 9A, high-risk ccRCC had more immune cell infiltration, such as T cell CD4 + central memory, T cell CD4+ Th1, myeloid dendritic cell activated and immune score at XCELL, macrophage M1 and monocyte at QUANTISEQ, cytotoxicity score at MCPCOUNTER, and T cell regulatory (Tregs) at CIBERSORT, some of which were visualized (Fig. 9B). Consistently, the high-risk group had a higher immune score (Fig. 9C). In the high-risk group, most of the immune checkpoints had a greater degree of activation (Fig. 9D). We then performed sensitivity analysis of immunotherapeutic agents and found that more than 40 immune agents had a lower IC50 in the high-risk cohort. Some of them were visualized, such as ABT.888 (Veliparib), a PARP inhibitor (Rimar et al. 2017) (Fig. 9E).

### Further differentiation of the immune microenvironment between ccRCC patients in clusters

**Fig. 10:**
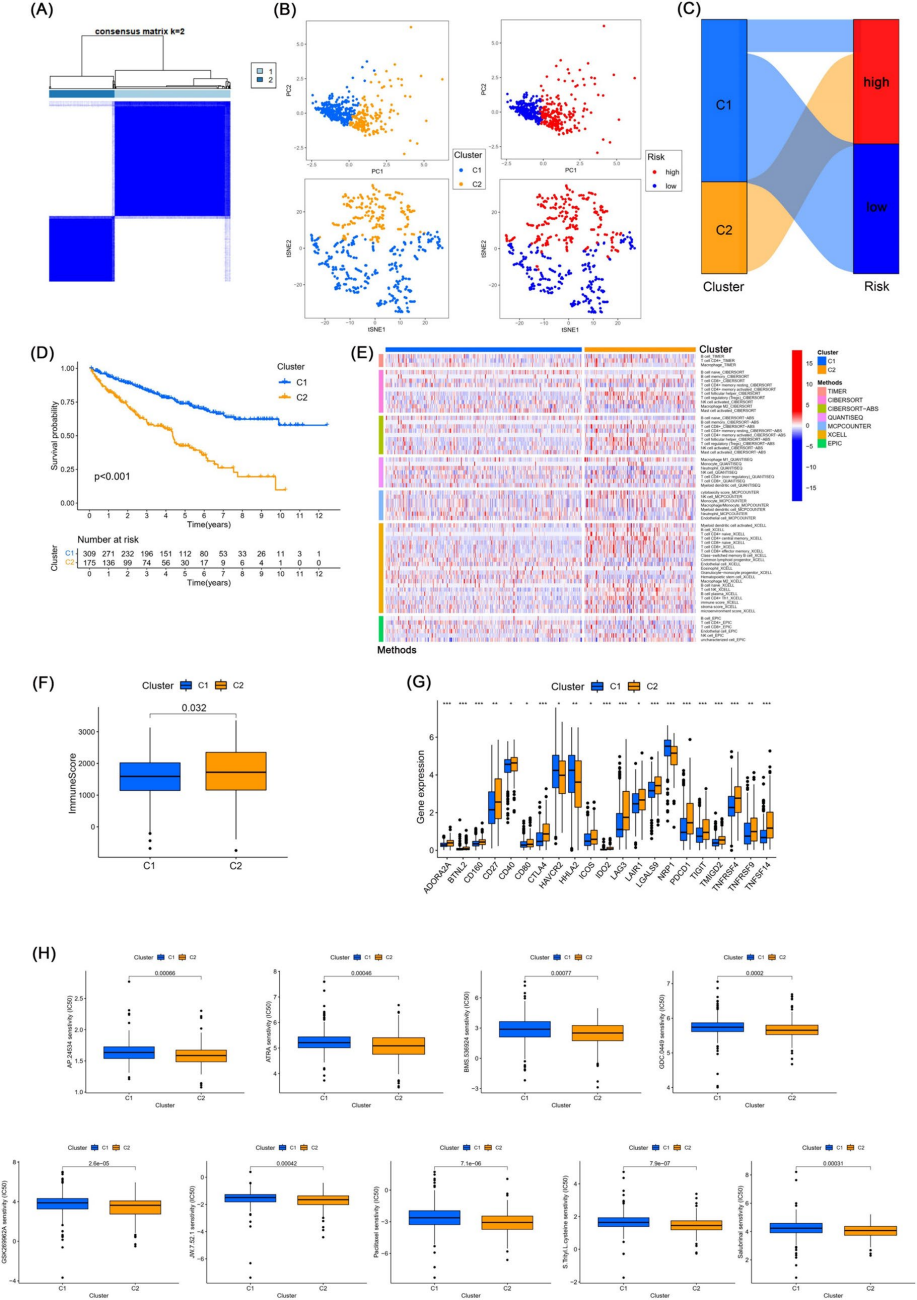
Clustering analysis and prediction for immunotherapy. **A** CcRCC patients in the TCGA database were reclassified into two clusters by consensus clustering analysis. **B** The principal component analysis (PCA) and t-distributed stochastic neighbor embedding (t-SNE) of risk groups and clusters. **C** The Sankey diagram demonstrated the relationship between risk groups and clusters. **D** Kaplan–Meier curves of overall survival between cluster 1 and cluster 2. Patients in cluster 2 have a worse prognosis. **E** The heatmap of immune infiltration levels in different platforms in two clusters. **F** Immune score in clusters. **G** Activation of immune checkpoints between cluster 1 and cluster 2, *p < 0.05, **p < 0.01, ***p < 0.001, ****p < 0.0001. **H** Immunotherapy sensitivity analysis of clusters

It has been shown that different subtypes of tumors, also called clusters, can have very different immune microenvironments (Xu et al. 2021). We, therefore, reclassified ccRCC patients into two clusters based on the expression of the three lncRNAs in the risk formula by consensus clustering (Fig. 10A). Principal component analyses (PCA) showed that the prognostic signature has a good ability to distinguish clusters and risk groups, and additionally t-distributed stochastic neighbor embedding (t-SNE) also demonstrated that clusters as well as risk groups could be clearly distinguished (Fig. 10B). The relationship between risk groups and clusters is shown in the Sankey diagram (Fig. 10C). Cluster 2 had a poor overall survival (*p* < 0.001) as shown by the Kaplan–Meier curve (Fig. 10D). We performed immune infiltration analysis and made a heatmap for both clusters based on different platforms, and the heatmap visualized that cluster 2 patients had more immune cell infiltration (Fig. 10E). The immune score was higher in cluster 2 than cluster 1 (*p* < 0.05) (Fig. 10F). Finally, we found that most of the immune checkpoints of cluster 2 were overactivated, such as CD27, CD40, CTLA-4, LAG-3, TIGIT, and TNFRSF4 (Fig. 10G). Therefore, we considered cluster 2 more likely to benefit in immunotherapy. In drug sensitivity analysis, we found that 52 immune agents had a lower IC50 in cluster 2, 9 of which had no significant differences in IC50 in risk groups (Fig. 10H).

## Discussion

Altered cyclin-dependent kinases (CDK) activity usually results in tumor-associated cell cycle defects, and although CDK-targeted therapeutic strategies have been considered to be therapeutically beneficial for some tumors, they are not the best therapeutic option considering that CDK is essential to drive every cell cycle phase in mammals (Malumbres and Barbacid 2009). CCNB2 regulates CDK activity by binding to CDK, thus affecting the normal division process of the cell cycle. In addition to this, CCNB2 transgenic mice are highly susceptible to tumorigenesis and CCNB2 is often overexpressed in human cancers (Nam and Deursen 2014). To date, there have been a number of studies on the role of CCNB2 in tumor progression. Liu et al. (2020) concluded that high CCNB2 expression was significantly associated with poor overall survival in hepatocellular carcinoma by analyzing data from multiple datasets. Gong et al. (2021) found that CCNB2 was associated with shorter overall survival in patients of non-small cell lung cancer (NSCLC) by bioinformatic analysis and confirmed the upregulation of CCNB2 in NSCLC tissues in cell experiments. To our knowledge, few studies have focused on exploring the effects of CCNB2 on prognostic indicators of ccRCC patients, such as DFS or OS. Therefore, in this study, we first analyzed the RNA sequences of ccRCC samples in TCGA and GEO databases by bioinformatics and evaluated the expression level and prognostic value of CCNB2 in ccRCC samples, and found that the expression of CCNB2 was significantly higher in ccRCC tissues than in normal tissues, and the high expression of CCNB2 was associated with a higher degree of malignancy of ccRCC. More importantly, the results of Kaplan–Meier curves and Cox regression analysis showed that high expression of CCNB2 was associated with poor survival in ccRCC patients and was an independent prognostic factor in ccRCC patients. These findings revealed that CCNB2 overexpression might affect ccRCC progression by disrupting the normal cell division process of the cell cycle.

The results of functional enrichment analysis and GSEA suggested that CCNB2 might play a role in ccRCC through cell division, mitochondrial spindle assembly checkpoint, E2F targets, G2M checkpoints, cell cycle, oocyte meiosis, and p53 signal pathway. Aneuploidy is one of the prominent phenotypes of cancer. When CCNB2 is overexpressed, CCNB2-CDK1 triggers aurora kinase A (AURKA) phosphorylation, which overactivates polo-like kinase 1 (PLK1) and subsequently accelerates centromere segregation, causing chromosomal lag and ultimately inducing the production of aneuploidy (Nam and Deursen 2014). Furthermore, p53 and CCNB2 are antagonistic for regulating centrosome segregation, suggesting that p53-deficient tumors are driven to develop through mechanisms involving abnormal centrosome segregation (Nam and Deursen 2014). In the cell cycle, mitosis is the stage most prone to aneuploidy errors, and mutations in the spindle assembly checkpoint (SAC) gene can lead to spontaneous tumors (Fang and Zhang 2011). Disruption of SAC contribute to cellular aneuploidy (Fang and Zhang 2011). P53 can be activated by aneuploidy or SAC defects, and highly aneuploid cells can overactivate p53 and thus die by apoptosis (Li et al. 2010; Thompson and Compton 2010). Retinoblastoma (RB) protein is a central regulator of the cell cycle, and RB binds to the E2F family of transcription factors to downregulate cell cycle gene transcription and suppress tumorigenesis by targeting cyclin, CDK, and others (Helin et al. 1993; Chicas et al. 2010; Fischer and Muller 2017).The RB pathway is usually inactivated in tumors, leading to dysregulation of E2F activity and promoting cell proliferation or death (Polager and Ginsberg 2008). In addition, it has been found that CCNB2 binds to CDK1 to produce the M phase-promoting factor (MPF), which is essential for the progression of meiosis in oocytes (Daldello et al. 2019). Sufficient pre-MPF needs to be prepared by CCNB2 to enable meiotic re-entry, and CCNB2 depletion leads to ovulation of immature oocytes and premature ovarian failure (Daldello et al. 2019). Therefore, although overexpression of cyclins is closely associated with tumor progression, it rescues the meiotic phenotype. Above studies were consistent with our results of pathway enrichment.

To enhance the objectivity of the study, we explored the immune cell infiltration of ccRCC using multiple modalities. Incredibly, we found that CCNB2 had the same expression pattern as a series of inhibitory immune checkpoints, such as PD-1, CTLA-4, and TIGIT. In addition to this, it was interesting to note that the expression of CCNB2 had a significant positive correlation with the level of infiltration of T regulatory cells (Tregs), myeloid-derived suppressor cells (MDSC). As we know, Tregs are the main contributors to the creation of suppressive tumor-immune microenvironment (Sharabi et al. 2018; Vuong et al. 2019). Also, recent research has suggested that IL-1beta may drive innate and adaptive immune resistance in ccRCC by promoting MDSC infiltration (Aggen et al. 2021). In addition, we found a significant positive correlation between CCNB2 expression and the infiltration of activated CD4 T cells, activated CD8 T cells, gamma delta T cells, Th2 cells, Tfh cells, and MDSCs in ccRCC. CD4+ T cells can differentiate into Tregs and multiple T helper (Th) cells, such as Th2 cells and Tfh cells. Th2 cells are associated with pro-tumor activity, enhancing angiogenesis, suppressing cellular immunity, and killing tumor cells. Some studies have shown evidence of the pro-tumor activity of Th2 cells in breast (Aspord et al. 2007) and colon cancer (Chen et al. 2018). Tfh cells are often thought to be involved in long-term humoral immunity by assisting B cells (Hetta et al. 2020). However, Tfh cells are associated with poor survival in an inflammation-induced mouse model of hepatocellular carcinoma (Crotty 2019). In addition, there are multiple subpopulations of CD8+ T cells, including Tc1, Tc2, Tc9, Tc17, and Tc22, not all of which are cytotoxic (St Paul and Ohashi 2020). Tc22 can exert pro-tumor activity by producing IL-22 in patients with transplantation-associated squamous carcinoma (Zhang et al. 2013). High Tc17 is implicated in poor prognosis in a variety of human cancers and may be related to its production of IL-17 (Zhuang et al. 2012; Wang et al. 2017). Gamma delta T cells, one of the unconventional subpopulations of T cells, have been reported to potentially exert immunosuppressive effects by hindering the proliferation of naïve T cells and the maturation of dendritic cells (Peng et al. 2007). It has also been shown that gamma delta T cells are the main source of IL-17 in colorectal cancer (Wu et al. 2014). IL-17 can promote angiogenesis and recruit MDSCs (St Paul and Ohashi 2020).

We then also mined the CCNB2-related miRNAs, lncRNAs regulatory network. First, we found four miRNAs (miR-23b-3p, miR-30a-5p, let-7c-3p, let-7c-5p) that were significantly and negatively correlated with CCNB2 expression and their low expression was associated with poor prognosis. We then explored the lncRNAs that interacted with these four miRNAs and found six lncRNAs (LINC00265, PVT1, SNHG17, VPS9D1-AS1, ZMIZ1-AS1, C3orf35) associated with poor prognosis, all of which were enriched in ccRCC tissue. We placed these survival-related lncRNAs into lasso regression analysis, and finally screened three lncRNAs (SNHG17, VPS9D1-AS1, ZMIZ1-AS1) to construct the risk formula. Patients were divided into high- and low-risk groups and underwent Kaplan–Meier analysis, immune cell infiltration analysis, and drug sensitivity analysis. We found that risk groups can provide guidance for predicting prognosis and immunotherapy. Considering that different molecular subtypes, also called clusters, can have very different immune microenvironments (DeBerardinis 2020), we subsequently divided all patients into two clusters by consensus clustering based on these lncRNAs. There were higher abundance of immune cell infiltration, higher immune score, and higher immune checkpoint activity in Cluster 2. In addition, we found that nine drugs showed differential IC50 in clusters, but not in risk groups. Overall, our inclusion of three genes provided a simpler prognostic formula capable of identifying the heat of tumor immunity, which might contribute to personalized treatment of ccRCC.

As we know, miRNA binds to the 3′-UTR of mRNA and thus negatively regulates mRNA expression, lncRNA can mediate miRNA sponging and thus change its function to produce pro-cancer effects (Su et al. 2021). MiR-30a-5p can inhibit breast tumor growth and metastasis by suppressing the Warburg effect (Li et al. 2017). Based on the results of functional enrichment and quantitative real-time polymerase chain reaction (qRT-PCR), Wu et al. (2020) proposed that the let-7c-5p/CCNB2 axis may be involved in the progression of cervical squamous carcinoma. Furthermore, there are also experimental results implying that the VPS9D1-AS1/miRNA-30a-5p axis promotes tumor malignant progression (Liu et al. 2021). SNHG17 has been reported to act as an oncogene that promotes tumor cell proliferation and migration while inhibiting apoptosis (Qin et al. 2020). Ma et al. published the first study involving SNHG17 and showed that SNHG17 contributed to tumor proliferation by epigenetically silencing P57 in colorectal cancer (Ma et al. 2017). In addition, Wu et al. found that SNHG17 may be involved in ccRCC progression by regulating H2AX signaling through miR-328-3p (Wu et al. 2021). Similarly, VPS9D1-AS1 has been shown to be overexpressed in several cancers, acts as a target of Wnt/c-Myc signaling and has pro-carcinogenic properties (Kawasaki et al. 2016). It was reported that the VPS9D1-AS1/hsa-miR-532-3p/BMP1 axis may be a potential regulatory pathway in ccRCC (Gong et al. 2023). In contrast, there are fewer reports on ZMIZ1-AS1. A study discovered that ZMIZ1-AS1 stabilized ZMIZ1 by recruiting the RNA-binding protein PTBP1. This promotes the proliferation and invasion of osteosarcoma (Zhou et al. 2022). Overall, the regulatory roles of lncRNAs and miRNAs on tumors are complex and multifaceted. Building models with survival-related lncRNAs can help us to cluster patients more accurately and bring breakthroughs for prognosis prediction and clinical treatment.

However, this study has some limitations. First, this study mainly focused on the analysis and validation at the level of big data, and despite a series of functional enrichment and signaling pathway analyses, basic research on the potential mechanisms of ccRCC signaling pathway was lacking. Second, although evaluated using many methods, our model still needs to be validated in real-world cohorts of ccRCC in the future. In the immune cell infiltration analysis, we showed the results of as many platforms as possible in the bubble plot and heatmap to try to achieve multifaceted validation.

## Conclusion

CCNB2 holds promise as a new target and prognostic marker in ccRCC treatment strategies, whose impact on prognosis may be related to tumorigenesis induced by abnormal cell division. In addition, a novel risk model was developed based on three CCNB2-related lncRNAs and two CCNB2-related molecular clusters were identified to improve personalized treatment in ccRCC patients.

### Supplementary Information

Below is the link to the electronic supplementary material.

Supplementary file1 (DOCX 15 KB)

Supplementary file2 (DOCX 42 KB)

## Data Availability

The datasets analyzed during the current study are available in TCGA repository (https://portal.gdc.cancer.gov), GEO (https://www.ncbi.nlm.nih.gov/geo/), GSEA (http://www.gsea-msigdb.org/gsea/index.jsp), and GDSC (https://www.cancerrxgene.org/).
