# Supplementary material for: Upregulation of CCNB2 and a novel lncRNAs-related risk model predict prognosis in clear cell renal cell carcinoma
Source: J Cancer Res Clin Oncol. 2024 Feb 1;150(2):64. doi: 10.1007/s00432-024-05611-x (PMC10834599; doi:10.1007/s00432-024-05611-x)
Supplement: Supplementary file 1 — Supplementary file1 (DOCX 15 KB) [file 432_2024_5611_MOESM1_ESM.docx]

**70 predicted miRNAs binding to CCNB2**

| **miRNA** | **Source** |
| --- | --- |
| hsa-miR-30d-5p | RNAInter |
| zikv-mir-9 | RNAInter |
| hsa-let-7b-5p | RNAInter |
| hsa-let-7f-5p | RNAInter |
| zikv-mir-7 | RNAInter |
| hsa-miR-30a-5p | RNAInter |
| hsa-miR-190b-5p | RNAInter |
| hsa-let-7c | RNAInter |
| hcmv-miR-US25-1-5p | RNAInter |
| jcv-miR-J1-5p | RNAInter |
| hsa-let-7a-1 | RNAInter |
| hsa-miR-23b-3p | RNAInter |
| hsa-miR-2681-5p | RNAInter |
| hsa-let-7a-5p | RNAInter |
| hsa-mir-23b | RNAInter |
| ebv-miR-BART22 | RNAInter |
| hsa-miR-126-3p | RNAInter |
| hsa-let-7c-5p | RNAInter |
| bkv-miR-B1-5p | RNAInter |
| zikv-mir-1 | RNAInter |
| hsa-let-7f-1 | RNAInter |
| kshv-miR-K12-3-5p | RNAInter |
| hsa-miR-582-3p | TargetScanHuman |
| hsa-miR-5197-3p | TargetScanHuman |
| hsa-miR-497-3p | TargetScanHuman |
| hsa-miR-548bb-3p | TargetScanHuman |
| hsa-miR-6847-5p | TargetScanHuman |
| hsa-miR-4315 | TargetScanHuman |
| hsa-miR-3653-3p | TargetScanHuman |
| hsa-miR-548ar-3p | TargetScanHuman |
| hsa-miR-548h-3p | TargetScanHuman |
| hsa-miR-6502-3p | TargetScanHuman |
| hsa-miR-195-3p | TargetScanHuman |
| hsa-miR-548az-3p | TargetScanHuman |
| hsa-miR-16-2-3p | TargetScanHuman |
| hsa-miR-548t-3p | TargetScanHuman |
| hsa-miR-548e-3p | TargetScanHuman |
| hsa-miR-548a-3p | TargetScanHuman |
| hsa-miR-548ac | TargetScanHuman |
| hsa-miR-6503-5p | TargetScanHuman |
| hsa-miR-1277-5p | TargetScanHuman |
| hsa-miR-4799-5p | TargetScanHuman |
| hsa-miR-3663-5p | TargetScanHuman |
| hsa-miR-6770-5p | TargetScanHuman |
| hsa-miR-4329 | TargetScanHuman |
| hsa-miR-1238-3p | TargetScanHuman |
| hsa-miR-4307 | TargetScanHuman |
| hsa-miR-6761-5p | TargetScanHuman |
| hsa-miR-3658 | TargetScanHuman |
| hsa-miR-7154-5p | TargetScanHuman |
| hsa-miR-548ap-3p | TargetScanHuman |
| hsa-miR-548aa | TargetScanHuman |
| hsa-miR-7107-3p | TargetScanHuman |
| hsa-miR-4790-5p | TargetScanHuman |
| hsa-miR-548f-3p | TargetScanHuman |
| hsa-miR-548z | TargetScanHuman |
| hsa-miR-4527 | TargetScanHuman |
| hsa-miR-8063 | TargetScanHuman |
| hsa-let-7c-3p | TargetScanHuman |
| hsa-miR-433-3p | TargetScanHuman |
| hsa-miR-548d-3p | TargetScanHuman |
| hsa-miR-551b-5p | TargetScanHuman |
| hsa-miR-1253 | TargetScanHuman |
| hsa-miR-6753-3p | TargetScanHuman |
| hsa-miR-4759 | TargetScanHuman |
| hsa-miR-335-5p | RNAInter&TargetScanHuman |
| hsa-miR-670-3p | RNAInter&TargetScanHuman |
| hsa-miR-6729-3p | RNAInter&TargetScanHuman |
| hsa-miR-3168 | RNAInter&TargetScanHuman |
| hsa-miR-4251 | RNAInter&TargetScanHuman |
