# Supplementary material for: Upregulation of CCNB2 and a novel lncRNAs-related risk model predict prognosis in clear cell renal cell carcinoma
Source: J Cancer Res Clin Oncol. 2024 Feb 1;150(2):64. doi: 10.1007/s00432-024-05611-x (PMC10834599; doi:10.1007/s00432-024-05611-x)
Supplement: Supplementary file 2 — Supplementary file2 (DOCX 42 KB) [file 432_2024_5611_MOESM2_ESM.docx]

**359 predicted lncRNAs binding to 4 CCNB2-related miRNAs**

| **lncRNA** | **miRNA** | **Source** |
| --- | --- | --- |
| ZNF436-AS1 | hsa-let-7c-5p | ENCORI |
| SNHG12 | hsa-let-7c-5p | ENCORI |
| AL050341.2 | hsa-let-7c-5p | ENCORI |
| AL360270.2 | hsa-let-7c-5p | ENCORI |
| AC239868.1 | hsa-let-7c-5p | ENCORI |
| AC234582.1 | hsa-let-7c-5p | ENCORI |
| AL590666.2 | hsa-let-7c-5p | ENCORI |
| MIR29B2CHG | hsa-let-7c-5p | ENCORI |
| AL359924.1 | hsa-let-7c-5p | ENCORI |
| AC074117.1 | hsa-let-7c-5p | ENCORI |
| LINC01806 | hsa-let-7c-5p | ENCORI |
| AC105760.2 | hsa-let-7c-5p | ENCORI |
| LMCD1-AS1 | hsa-let-7c-5p | ENCORI |
| AC124045.1 | hsa-let-7c-5p | ENCORI |
| CCDC37-AS1 | hsa-let-7c-5p | ENCORI |
| AC097103.2 | hsa-let-7c-5p | ENCORI |
| MUC20-OT1 | hsa-let-7c-5p | ENCORI |
| LINC00885 | hsa-let-7c-5p | ENCORI |
| LINC02432 | hsa-let-7c-5p | ENCORI |
| AC093908.1 | hsa-let-7c-5p | ENCORI |
| SLC9A3-AS1 | hsa-let-7c-5p | ENCORI |
| LINC02242 | hsa-let-7c-5p | ENCORI |
| SNHG4 | hsa-let-7c-5p | ENCORI |
| CARMN | hsa-let-7c-5p | ENCORI |
| HEIH | hsa-let-7c-5p | ENCORI |
| AC138035.1 | hsa-let-7c-5p | ENCORI |
| AL024498.1 | hsa-let-7c-5p | ENCORI |
| HCG18 | hsa-let-7c-5p | ENCORI |
| IER3-AS1 | hsa-let-7c-5p | ENCORI |
| AC080080.1 | hsa-let-7c-5p | ENCORI |
| AC004520.1 | hsa-let-7c-5p | ENCORI |
| HOXA11-AS | hsa-let-7c-5p | ENCORI |
| TRG-AS1 | hsa-let-7c-5p | ENCORI |
| LINC00265 | hsa-let-7c-5p | ENCORI |
| AC073335.2 | hsa-let-7c-5p | ENCORI |
| AC004921.1 | hsa-let-7c-5p | ENCORI |
| STAG3L5P-PVRIG2P-PILRB | hsa-let-7c-5p | ENCORI |
| AC023632.6 | hsa-let-7c-5p | ENCORI |
| CDKN2B-AS1 | hsa-let-7c-5p | ENCORI |
| AL590705.3 | hsa-let-7c-5p | ENCORI |
| LINC00963 | hsa-let-7c-5p | ENCORI |
| AL157392.3 | hsa-let-7c-5p | ENCORI |
| AC010997.4 | hsa-let-7c-5p | ENCORI |
| AL132656.4 | hsa-let-7c-5p | ENCORI |
| NUTM2A-AS1 | hsa-let-7c-5p | ENCORI |
| OLMALINC | hsa-let-7c-5p | ENCORI |
| RPARP-AS1 | hsa-let-7c-5p | ENCORI |
| LINC01001 | hsa-let-7c-5p | ENCORI |
| AC240565.2 | hsa-let-7c-5p | ENCORI |
| KCNQ1OT1 | hsa-let-7c-5p | ENCORI |
| LINC00294 | hsa-let-7c-5p | ENCORI |
| NEAT1 | hsa-let-7c-5p | ENCORI |
| AP002360.3 | hsa-let-7c-5p | ENCORI |
| AP000873.2 | hsa-let-7c-5p | ENCORI |
| AP000446.1 | hsa-let-7c-5p | ENCORI |
| AP000766.1 | hsa-let-7c-5p | ENCORI |
| AC125807.2 | hsa-let-7c-5p | ENCORI |
| AC006206.1 | hsa-let-7c-5p | ENCORI |
| AC006064.5 | hsa-let-7c-5p | ENCORI |
| AC022075.1 | hsa-let-7c-5p | ENCORI |
| AC009318.1 | hsa-let-7c-5p | ENCORI |
| AC125611.4 | hsa-let-7c-5p | ENCORI |
| AC012531.2 | hsa-let-7c-5p | ENCORI |
| LINC02381 | hsa-let-7c-5p | ENCORI |
| AC048341.1 | hsa-let-7c-5p | ENCORI |
| AC090001.1 | hsa-let-7c-5p | ENCORI |
| TMPO-AS1 | hsa-let-7c-5p | ENCORI |
| HELLPAR | hsa-let-7c-5p | ENCORI |
| AC026362.1 | hsa-let-7c-5p | ENCORI |
| AC122688.3 | hsa-let-7c-5p | ENCORI |
| AL136419.1 | hsa-let-7c-5p | ENCORI |
| G2E3-AS1 | hsa-let-7c-5p | ENCORI |
| AL445363.3 | hsa-let-7c-5p | ENCORI |
| AL049780.3 | hsa-let-7c-5p | ENCORI |
| AF111167.2 | hsa-let-7c-5p | ENCORI |
| VASH1-AS1 | hsa-let-7c-5p | ENCORI |
| MEG8 | hsa-let-7c-5p | ENCORI |
| AL117190.1 | hsa-let-7c-5p | ENCORI |
| OIP5-AS1 | hsa-let-7c-5p | ENCORI |
| AC055855.1 | hsa-let-7c-5p | ENCORI |
| IQCH-AS1 | hsa-let-7c-5p | ENCORI |
| DRAIC | hsa-let-7c-5p | ENCORI |
| THSD4-AS1 | hsa-let-7c-5p | ENCORI |
| UBL7-AS1 | hsa-let-7c-5p | ENCORI |
| AC105020.1 | hsa-let-7c-5p | ENCORI |
| AC090825.1 | hsa-let-7c-5p | ENCORI |
| AC015712.2 | hsa-let-7c-5p | ENCORI |
| AC009065.3 | hsa-let-7c-5p | ENCORI |
| AC109460.3 | hsa-let-7c-5p | ENCORI |
| AC106886.4 | hsa-let-7c-5p | ENCORI |
| AC026471.1 | hsa-let-7c-5p | ENCORI |
| AC020978.6 | hsa-let-7c-5p | ENCORI |
| AC020978.5 | hsa-let-7c-5p | ENCORI |
| AC009022.1 | hsa-let-7c-5p | ENCORI |
| AC012184.3 | hsa-let-7c-5p | ENCORI |
| AC092139.3 | hsa-let-7c-5p | ENCORI |
| AC133540.1 | hsa-let-7c-5p | ENCORI |
| AC005224.4 | hsa-let-7c-5p | ENCORI |
| AC093484.1 | hsa-let-7c-5p | ENCORI |
| ARHGAP27P1-BPTFP1-KPNA2P3 | hsa-let-7c-5p | ENCORI |
| AC037487.4 | hsa-let-7c-5p | ENCORI |
| SNHG16 | hsa-let-7c-5p | ENCORI |
| LINC01978 | hsa-let-7c-5p | ENCORI |
| AC124319.4 | hsa-let-7c-5p | ENCORI |
| AC007996.1 | hsa-let-7c-5p | ENCORI |
| AC116447.1 | hsa-let-7c-5p | ENCORI |
| AC091060.1 | hsa-let-7c-5p | ENCORI |
| AC011447.7 | hsa-let-7c-5p | ENCORI |
| TMEM147-AS1 | hsa-let-7c-5p | ENCORI |
| LINC00665 | hsa-let-7c-5p | ENCORI |
| ZNF571-AS1 | hsa-let-7c-5p | ENCORI |
| AC006486.2 | hsa-let-7c-5p | ENCORI |
| AC243964.3 | hsa-let-7c-5p | ENCORI |
| AC010327.5 | hsa-let-7c-5p | ENCORI |
| AC007228.2 | hsa-let-7c-5p | ENCORI |
| AC020915.3 | hsa-let-7c-5p | ENCORI |
| ZNF337-AS1 | hsa-let-7c-5p | ENCORI |
| AL121845.1 | hsa-let-7c-5p | ENCORI |
| AF254983.1 | hsa-let-7c-5p | ENCORI |
| MIR99AHG | hsa-let-7c-5p | ENCORI |
| LINC01678 | hsa-let-7c-5p | ENCORI |
| TTC28-AS1 | hsa-let-7c-5p | ENCORI |
| AC005005.4 | hsa-let-7c-5p | ENCORI |
| MIRLET7BHG | hsa-let-7c-5p | ENCORI |
| AC232271.1 | hsa-let-7c-5p | ENCORI |
| XIST | hsa-let-7c-5p | ENCORI |
| LINC00894 | hsa-let-7c-5p | ENCORI |
| TTTY15 | hsa-let-7c-5p | ENCORI |
| LAMTOR5-AS1 | hsa-miR-23b-3p | ENCORI |
| AC242426.2 | hsa-miR-23b-3p | ENCORI |
| AC239868.1 | hsa-miR-23b-3p | ENCORI |
| GAS5 | hsa-miR-23b-3p | ENCORI |
| AL078645.1 | hsa-miR-23b-3p | ENCORI |
| DNAJC27-AS1 | hsa-miR-23b-3p | ENCORI |
| LINC01122 | hsa-miR-23b-3p | ENCORI |
| AC133644.3 | hsa-miR-23b-3p | ENCORI |
| AC068282.1 | hsa-miR-23b-3p | ENCORI |
| LINC01876 | hsa-miR-23b-3p | ENCORI |
| TTN-AS1 | hsa-miR-23b-3p | ENCORI |
| LINC01963 | hsa-miR-23b-3p | ENCORI |
| AC016717.2 | hsa-miR-23b-3p | ENCORI |
| AC005538.2 | hsa-miR-23b-3p | ENCORI |
| THUMPD3-AS1 | hsa-miR-23b-3p | ENCORI |
| THRB-IT1 | hsa-miR-23b-3p | ENCORI |
| PTPRG-AS1 | hsa-miR-23b-3p | ENCORI |
| LINC02035 | hsa-miR-23b-3p | ENCORI |
| AC069528.2 | hsa-miR-23b-3p | ENCORI |
| LINC01995 | hsa-miR-23b-3p | ENCORI |
| AC022498.2 | hsa-miR-23b-3p | ENCORI |
| AC139887.2 | hsa-miR-23b-3p | ENCORI |
| AL136537.2 | hsa-miR-23b-3p | ENCORI |
| AC107068.1 | hsa-miR-23b-3p | ENCORI |
| AC093895.1 | hsa-miR-23b-3p | ENCORI |
| AC097478.1 | hsa-miR-23b-3p | ENCORI |
| MIR4453HG | hsa-miR-23b-3p | ENCORI |
| AC105285.1 | hsa-miR-23b-3p | ENCORI |
| RP11-395P13.2 | hsa-miR-23b-3p | ENCORI |
| MALINC1 | hsa-miR-23b-3p | ENCORI |
| LINC01485 | hsa-miR-23b-3p | ENCORI |
| ZSCAN16-AS1 | hsa-miR-23b-3p | ENCORI |
| LINC00472 | hsa-miR-23b-3p | ENCORI |
| SNHG5 | hsa-miR-23b-3p | ENCORI |
| TRAF3IP2-AS1 | hsa-miR-23b-3p | ENCORI |
| AL024508.2 | hsa-miR-23b-3p | ENCORI |
| LINC00473 | hsa-miR-23b-3p | ENCORI |
| TRG-AS1 | hsa-miR-23b-3p | ENCORI |
| MAGI2-AS3 | hsa-miR-23b-3p | ENCORI |
| AC000120.1 | hsa-miR-23b-3p | ENCORI |
| STAG3L5P-PVRIG2P-PILRB | hsa-miR-23b-3p | ENCORI |
| ST7-OT4 | hsa-miR-23b-3p | ENCORI |
| AC078846.1 | hsa-miR-23b-3p | ENCORI |
| AF131215.4 | hsa-miR-23b-3p | ENCORI |
| AC079193.2 | hsa-miR-23b-3p | ENCORI |
| MSC-AS1 | hsa-miR-23b-3p | ENCORI |
| AC090198.1 | hsa-miR-23b-3p | ENCORI |
| PVT1 | hsa-miR-23b-3p | ENCORI |
| AL162231.2 | hsa-miR-23b-3p | ENCORI |
| ZEB1-AS1 | hsa-miR-23b-3p | ENCORI |
| ZMIZ1-AS1 | hsa-miR-23b-3p | ENCORI |
| AL132656.2 | hsa-miR-23b-3p | ENCORI |
| NUTM2B-AS1 | hsa-miR-23b-3p | ENCORI |
| OLMALINC | hsa-miR-23b-3p | ENCORI |
| AL133215.2 | hsa-miR-23b-3p | ENCORI |
| AL133355.1 | hsa-miR-23b-3p | ENCORI |
| AC091564.7 | hsa-miR-23b-3p | ENCORI |
| SNHG1 | hsa-miR-23b-3p | ENCORI |
| NEAT1 | hsa-miR-23b-3p | ENCORI |
| MALAT1 | hsa-miR-23b-3p | ENCORI |
| AP003419.2 | hsa-miR-23b-3p | ENCORI |
| AP002360.3 | hsa-miR-23b-3p | ENCORI |
| AP006216.1 | hsa-miR-23b-3p | ENCORI |
| AC010168.2 | hsa-miR-23b-3p | ENCORI |
| AC048344.4 | hsa-miR-23b-3p | ENCORI |
| AC026356.1 | hsa-miR-23b-3p | ENCORI |
| AC068888.1 | hsa-miR-23b-3p | ENCORI |
| LINC01481 | hsa-miR-23b-3p | ENCORI |
| AC144548.1 | hsa-miR-23b-3p | ENCORI |
| LINC00173 | hsa-miR-23b-3p | ENCORI |
| AC073857.1 | hsa-miR-23b-3p | ENCORI |
| AC055713.1 | hsa-miR-23b-3p | ENCORI |
| LINC00943 | hsa-miR-23b-3p | ENCORI |
| AC073911.2 | hsa-miR-23b-3p | ENCORI |
| AL355001.2 | hsa-miR-23b-3p | ENCORI |
| N4BP2L2-IT2 | hsa-miR-23b-3p | ENCORI |
| DLEU2 | hsa-miR-23b-3p | ENCORI |
| AL096870.2 | hsa-miR-23b-3p | ENCORI |
| KTN1-AS1 | hsa-miR-23b-3p | ENCORI |
| AL133370.1 | hsa-miR-23b-3p | ENCORI |
| AL136040.1 | hsa-miR-23b-3p | ENCORI |
| AL121839.2 | hsa-miR-23b-3p | ENCORI |
| AL163051.1 | hsa-miR-23b-3p | ENCORI |
| AL163051.2 | hsa-miR-23b-3p | ENCORI |
| LINC01550 | hsa-miR-23b-3p | ENCORI |
| MEG3 | hsa-miR-23b-3p | ENCORI |
| MEG8 | hsa-miR-23b-3p | ENCORI |
| AL583810.1 | hsa-miR-23b-3p | ENCORI |
| AC127502.2 | hsa-miR-23b-3p | ENCORI |
| AC020661.1 | hsa-miR-23b-3p | ENCORI |
| OIP5-AS1 | hsa-miR-23b-3p | ENCORI |
| EIF3J-AS1 | hsa-miR-23b-3p | ENCORI |
| GABPB1-AS1 | hsa-miR-23b-3p | ENCORI |
| AC068338.2 | hsa-miR-23b-3p | ENCORI |
| AC124068.2 | hsa-miR-23b-3p | ENCORI |
| AC104035.1 | hsa-miR-23b-3p | ENCORI |
| LINC01579 | hsa-miR-23b-3p | ENCORI |
| AC015712.2 | hsa-miR-23b-3p | ENCORI |
| AC040162.3 | hsa-miR-23b-3p | ENCORI |
| AC009022.1 | hsa-miR-23b-3p | ENCORI |
| AC079414.3 | hsa-miR-23b-3p | ENCORI |
| AC009113.1 | hsa-miR-23b-3p | ENCORI |
| VPS9D1-AS1 | hsa-miR-23b-3p | ENCORI |
| AC002347.1 | hsa-miR-23b-3p | ENCORI |
| AC015922.4 | hsa-miR-23b-3p | ENCORI |
| RP1-178F10.3 | hsa-miR-23b-3p | ENCORI |
| AC026271.3 | hsa-miR-23b-3p | ENCORI |
| AC015726.1 | hsa-miR-23b-3p | ENCORI |
| CTD-2132N18.4 | hsa-miR-23b-3p | ENCORI |
| TOB1-AS1 | hsa-miR-23b-3p | ENCORI |
| AC018628.1 | hsa-miR-23b-3p | ENCORI |
| AC005332.6 | hsa-miR-23b-3p | ENCORI |
| AC007780.1 | hsa-miR-23b-3p | ENCORI |
| SNHG16 | hsa-miR-23b-3p | ENCORI |
| AC145207.5 | hsa-miR-23b-3p | ENCORI |
| TYMSOS | hsa-miR-23b-3p | ENCORI |
| LINC00909 | hsa-miR-23b-3p | ENCORI |
| PARD6G-AS1 | hsa-miR-23b-3p | ENCORI |
| AC010636.2 | hsa-miR-23b-3p | ENCORI |
| AC092329.4 | hsa-miR-23b-3p | ENCORI |
| AC011503.2 | hsa-miR-23b-3p | ENCORI |
| AC008736.1 | hsa-miR-23b-3p | ENCORI |
| AC243960.1 | hsa-miR-23b-3p | ENCORI |
| CTD-2537I9.5 | hsa-miR-23b-3p | ENCORI |
| AC012313.3 | hsa-miR-23b-3p | ENCORI |
| LINC01727 | hsa-miR-23b-3p | ENCORI |
| LINC00261 | hsa-miR-23b-3p | ENCORI |
| AL110115.1 | hsa-miR-23b-3p | ENCORI |
| AL035458.2 | hsa-miR-23b-3p | ENCORI |
| SNHG17 | hsa-miR-23b-3p | ENCORI |
| AF127936.2 | hsa-miR-23b-3p | ENCORI |
| MCM3AP-AS1 | hsa-miR-23b-3p | ENCORI |
| AC004019.18 | hsa-miR-23b-3p | ENCORI |
| Z95115.1 | hsa-miR-23b-3p | ENCORI |
| Z93930.2 | hsa-miR-23b-3p | ENCORI |
| Z93241.1 | hsa-miR-23b-3p | ENCORI |
| Z95331.1 | hsa-miR-23b-3p | ENCORI |
| ZNF674-AS1 | hsa-miR-23b-3p | ENCORI |
| XIST | hsa-miR-23b-3p | ENCORI |
| AL391244.1 | hsa-miR-30a-5p | ENCORI |
| AC254633.1 | hsa-miR-30a-5p | ENCORI |
| LINC01355 | hsa-miR-30a-5p | ENCORI |
| AC239868.1 | hsa-miR-30a-5p | ENCORI |
| LINC01133 | hsa-miR-30a-5p | ENCORI |
| RP11-226L15.5 | hsa-miR-30a-5p | ENCORI |
| LINC01139 | hsa-miR-30a-5p | ENCORI |
| AL390728.6 | hsa-miR-30a-5p | ENCORI |
| AL133243.3 | hsa-miR-30a-5p | ENCORI |
| AC016747.1 | hsa-miR-30a-5p | ENCORI |
| AC005034.3 | hsa-miR-30a-5p | ENCORI |
| AC092168.2 | hsa-miR-30a-5p | ENCORI |
| PAX8-AS1 | hsa-miR-30a-5p | ENCORI |
| AC016717.2 | hsa-miR-30a-5p | ENCORI |
| AC010148.1 | hsa-miR-30a-5p | ENCORI |
| AC069222.1 | hsa-miR-30a-5p | ENCORI |
| ACAP2-IT1 | hsa-miR-30a-5p | ENCORI |
| CTBP1-AS2 | hsa-miR-30a-5p | ENCORI |
| NOP14-AS1 | hsa-miR-30a-5p | ENCORI |
| AC109347.1 | hsa-miR-30a-5p | ENCORI |
| LIFR-AS1 | hsa-miR-30a-5p | ENCORI |
| AC008966.1 | hsa-miR-30a-5p | ENCORI |
| EPB41L4A-AS1 | hsa-miR-30a-5p | ENCORI |
| AC021078.1 | hsa-miR-30a-5p | ENCORI |
| AL137003.2 | hsa-miR-30a-5p | ENCORI |
| AL353759.1 | hsa-miR-30a-5p | ENCORI |
| AL662795.1 | hsa-miR-30a-5p | ENCORI |
| HCG18 | hsa-miR-30a-5p | ENCORI |
| LINC02535 | hsa-miR-30a-5p | ENCORI |
| AL096817.1 | hsa-miR-30a-5p | ENCORI |
| AL024507.2 | hsa-miR-30a-5p | ENCORI |
| AC018648.1 | hsa-miR-30a-5p | ENCORI |
| AC073335.2 | hsa-miR-30a-5p | ENCORI |
| SLC26A4-AS1 | hsa-miR-30a-5p | ENCORI |
| AC144652.1 | hsa-miR-30a-5p | ENCORI |
| AF131215.3 | hsa-miR-30a-5p | ENCORI |
| AP006248.4 | hsa-miR-30a-5p | ENCORI |
| AC009812.1 | hsa-miR-30a-5p | ENCORI |
| AF117829.1 | hsa-miR-30a-5p | ENCORI |
| AC023632.6 | hsa-miR-30a-5p | ENCORI |
| PVT1 | hsa-miR-30a-5p | ENCORI |
| LINC00707 | hsa-miR-30a-5p | ENCORI |
| AL139125.2 | hsa-miR-30a-5p | ENCORI |
| GATA3-AS1 | hsa-miR-30a-5p | ENCORI |
| AL157392.3 | hsa-miR-30a-5p | ENCORI |
| C10orf25 | hsa-miR-30a-5p | ENCORI |
| AC091769.2 | hsa-miR-30a-5p | ENCORI |
| AC010997.6 | hsa-miR-30a-5p | ENCORI |
| NUTM2B-AS1 | hsa-miR-30a-5p | ENCORI |
| DNMBP-AS1 | hsa-miR-30a-5p | ENCORI |
| AL158835.1 | hsa-miR-30a-5p | ENCORI |
| AL158835.4 | hsa-miR-30a-5p | ENCORI |
| KRTAP5-AS1 | hsa-miR-30a-5p | ENCORI |
| KCNQ1OT1 | hsa-miR-30a-5p | ENCORI |
| NEAT1 | hsa-miR-30a-5p | ENCORI |
| AP000769.2 | hsa-miR-30a-5p | ENCORI |
| MALAT1 | hsa-miR-30a-5p | ENCORI |
| AP000577.1 | hsa-miR-30a-5p | ENCORI |
| PCF11-AS1 | hsa-miR-30a-5p | ENCORI |
| AP000766.1 | hsa-miR-30a-5p | ENCORI |
| AC026356.1 | hsa-miR-30a-5p | ENCORI |
| AC008124.1 | hsa-miR-30a-5p | ENCORI |
| LINC01481 | hsa-miR-30a-5p | ENCORI |
| HELLPAR | hsa-miR-30a-5p | ENCORI |
| MAPKAPK5-AS1 | hsa-miR-30a-5p | ENCORI |
| LINC01089 | hsa-miR-30a-5p | ENCORI |
| AC137767.1 | hsa-miR-30a-5p | ENCORI |
| AC055713.1 | hsa-miR-30a-5p | ENCORI |
| AL512506.2 | hsa-miR-30a-5p | ENCORI |
| DLEU2 | hsa-miR-30a-5p | ENCORI |
| AL442125.1 | hsa-miR-30a-5p | ENCORI |
| AL445363.3 | hsa-miR-30a-5p | ENCORI |
| PSMA3-AS1 | hsa-miR-30a-5p | ENCORI |
| AL137129.1 | hsa-miR-30a-5p | ENCORI |
| AL162171.3 | hsa-miR-30a-5p | ENCORI |
| AL122023.1 | hsa-miR-30a-5p | ENCORI |
| AL049840.4 | hsa-miR-30a-5p | ENCORI |
| AC124312.4 | hsa-miR-30a-5p | ENCORI |
| PWAR5 | hsa-miR-30a-5p | ENCORI |
| AC012236.1 | hsa-miR-30a-5p | ENCORI |
| OIP5-AS1 | hsa-miR-30a-5p | ENCORI |
| AC084757.2 | hsa-miR-30a-5p | ENCORI |
| DNAAF4-CCPG1 | hsa-miR-30a-5p | ENCORI |
| IQCH-AS1 | hsa-miR-30a-5p | ENCORI |
| AC009269.5 | hsa-miR-30a-5p | ENCORI |
| ST20-AS1 | hsa-miR-30a-5p | ENCORI |
| AC016705.2 | hsa-miR-30a-5p | ENCORI |
| AL031009.1 | hsa-miR-30a-5p | ENCORI |
| LINC00921 | hsa-miR-30a-5p | ENCORI |
| AC092718.7 | hsa-miR-30a-5p | ENCORI |
| AC107983.2 | hsa-miR-30a-5p | ENCORI |
| AC138150.2 | hsa-miR-30a-5p | ENCORI |
| TBC1D3P1-DHX40P1 | hsa-miR-30a-5p | ENCORI |
| AC018628.1 | hsa-miR-30a-5p | ENCORI |
| AC005332.6 | hsa-miR-30a-5p | ENCORI |
| SOX9-AS1 | hsa-miR-30a-5p | ENCORI |
| SNHG16 | hsa-miR-30a-5p | ENCORI |
| AC145207.5 | hsa-miR-30a-5p | ENCORI |
| AC132938.5 | hsa-miR-30a-5p | ENCORI |
| GACAT2 | hsa-miR-30a-5p | ENCORI |
| AC018529.3 | hsa-miR-30a-5p | ENCORI |
| RAB11B-AS1 | hsa-miR-30a-5p | ENCORI |
| AC020910.5 | hsa-miR-30a-5p | ENCORI |
| TMEM147-AS1 | hsa-miR-30a-5p | ENCORI |
| ZNF571-AS1 | hsa-miR-30a-5p | ENCORI |
| PTOV1-AS1 | hsa-miR-30a-5p | ENCORI |
| AL109935.1 | hsa-miR-30a-5p | ENCORI |
| LINC00261 | hsa-miR-30a-5p | ENCORI |
| NORAD | hsa-miR-30a-5p | ENCORI |
| GUSBP11 | hsa-miR-30a-5p | ENCORI |
| AP000347.2 | hsa-miR-30a-5p | ENCORI |
| LINC00899 | hsa-miR-30a-5p | ENCORI |
| XIST | hsa-miR-30a-5p | ENCORI |
| AL035425.3 | hsa-miR-30a-5p | ENCORI |
| H19 | hsa-let-7c-3p | RNAInter |
| SNHG16 | hsa-let-7c-3p | RNAInter |
| PVT1 | hsa-let-7c-3p | RNAInter |
| LINC-ROR | hsa-let-7c-3p | RNAInter |
| CCAT1 | hsa-let-7c-3p | RNAInter |
| AC106038.1 | hsa-let-7c-3p | RNAInter |
| AC016355.1 | hsa-let-7c-3p | RNAInter |
| C17orf77 | hsa-let-7c-3p | TargetScanHuman |
| C5orf64 | hsa-let-7c-3p | TargetScanHuman |
| C2orf91 | hsa-let-7c-3p | TargetScanHuman |
| SLC25A21-AS1 | hsa-let-7c-3p | TargetScanHuman |
| FAM230A | hsa-let-7c-3p | TargetScanHuman |
| AC010536.1 | hsa-let-7c-3p | TargetScanHuman |
| COLCA1 | hsa-let-7c-3p | TargetScanHuman |
| KIAA0087 | hsa-let-7c-3p | TargetScanHuman |
| LINC00955 | hsa-let-7c-3p | TargetScanHuman |
| AP000708.1 | hsa-let-7c-3p | TargetScanHuman |
| C6orf223 | hsa-let-7c-3p | TargetScanHuman |
| PAXIP1-AS2 | hsa-let-7c-3p | TargetScanHuman |
| ST7-OT4 | hsa-let-7c-3p | TargetScanHuman |
| C3orf35 | hsa-let-7c-3p | TargetScanHuman |
